# The influence of hypertensive disorders in pregnancy on neonatal amino acid and acylcarnitine levels

**DOI:** 10.3389/fnut.2025.1520262

**Published:** 2025-08-15

**Authors:** Shiyi Xu, Fei Kong, Shuting Huang, Qiuping Liao, Jinfu Zhou, Jinying Luo

**Affiliations:** ^1^Fujian Maternity and Child Hospital College of Clinical Medicine for Obstetrics and Gynecology and Pediatrics, Fujian Medical University, Fuzhou, China; ^2^Department of Preventive Medicine, School of Public Health, Fujian Medical University, Fuzhou, China; ^3^Department of Obstetrics and Gynecology, Fujian Maternity and Child Health Hospital, College of Clinical Medicine for Obstetrics and Gynecology and Pediatrics, Fujian Medical University, Fuzhou, China; ^4^Medical Genetic Diagnosis and Therapy Center, Fujian Maternity and Child Hospital College of Clinical Medicine for Obstetrics and Gynecology and Pediatrics, Fujian Medical University, Fuzhou, China; ^5^Fujian Provincial Key Laboratory for Prenatal Diagnosis and Birth Defect, Fuzhou, China

**Keywords:** hypertensive disorders in pregnancy, pre-eclampsia, gestational hypertension, neonatal metabolism, amino acids, acylcarnitines

## Abstract

**Background/objectives:**

Hypertensive disorders in pregnancy (HDP) are associated with an increased risk of neonatal complications; however, their effects on neonatal metabolism remain inadequately understood. The aim of this study was to assess the association between HDP and neonatal amino acid and acylcarnitine levels.

**Methods:**

In this retrospective case–control study, 1,228 singleton pregnant women diagnosed with HDP and 1,228 normal singleton pregnant women whose newborns underwent newborn screening for 11 amino acids and 31 acylcarnitines were recruited from January 2021 to December 2023.

**Results:**

After adjusting for potential confounding factors, including gestational age at delivery, birth weight and neonatal sex, nine amino acids exhibited significant differences between infants born to mothers in the HDP subgroups compared to those born to mothers with normal pregnancies. These amino acids were involved in arginine and proline metabolism and the urea cycle pathway. Amino acid levels also varied among the HDP subgroups. Additionally, the levels of short-, medium-, and long-chain acylcarnitines were significantly higher in newborns born to mothers in the HDP subgroups than in newborns born to mothers in the normal pregnancy group. However, no statistically significant differences were observed among the four HDP subgroups.

**Conclusion:**

Our findings revealed a significant link between HDP and neonatal amino acid and acylcarnitine levels, which were involved in arginine and proline metabolism, the urea cycle, and fatty acid oxidation. These results underscore the significance of identifying maternal conditions that affect newborn metabolites to ensure adequate nutrition and enhance neonatal health outcomes.

## 1 Introduction

Hypertensive disorders in pregnancy (HDP) are a leading cause of maternal and perinatal deaths worldwide and classified into four main categories: gestational hypertension (GH), pre-eclampsia-eclampsia (PE), chronic hypertension (CH), and chronic hypertension with superimposed pre-eclampsia (CH + PE) (1–3). HDP affects 5–15% of all pregnancies and is among the most common prenatal complications, ranking second as the leading cause of maternal death globally, following maternal hemorrhage (4). From 2017 to 2019, the prevalence of HDP among delivery hospitalizations in the United States increased from 13.3 to 15.9% (5). Among deaths that occurred during delivery hospitalization, 31.6% had a documentation of HDP. In addition to maternal risks, HDP is also associated with adverse fetal outcomes, such as preterm labor, fetal growth restriction (FGR), and stillbirth (6–8). The weighted stillbirth rate in women with HDP in China from 2012 to 2019 was 21.9 per 1,000 births (9). In addition, the unfavorable in utero conditions associated with HDP may increase the risk of long-term vascular, cognitive, and psychiatric sequelae in offspring (10–14).

Studies have shown that pregnancy disorders and prenatal exposure to pollutants can alter the metabolic profiles of offspring (15–18), significantly influencing their long-term metabolic health and neurodevelopment (19–22). Newborn screening (NBS) is a population-wide test designed to identify inborn metabolic errors through the analysis of neonatal amino acid and acylcarnitine levels. These molecular phenotypic changes reflect not only hereditary metabolic disorders but also altered metabolic states associated with clinical conditions unrelated to primary genetic or metabolic diagnoses. Both essential and nonessential amino acids, as well as acetyl carnitines, regulate key metabolic pathways to improve fetal development during pregnancy (23, 24). Some of the nonessential amino acids (e.g., glutamine, glutamate, and arginine) play important roles in regulating gene expression, cell signaling, antioxidant responses, immunity, and neurological function (24). Disruptions in maternal amino acid metabolism, resulting in fetal metabolism disturbance, have been associated with various adult diseases later in life, a phenomenon referred to as the developmental origins of health and disease (25, 26).

Furthermore, studies have demonstrated that NBS can provide significant insights into metabolic disturbances related to factors beyond genetic inborn metabolic errors, including birth weight, gestational age (27), and the risk of acquired diseases in newborns such as sepsis (28), necrotizing enterocolitis (29), hyperbilirubinemia (30), persistent pulmonary hypertension (31), hypoxic–ischemic encephalopathy (32), and mortality (33). However, the influence of maternal pregnancy complications and comorbidities on newborn metabolic function, as indicated by NBS, has not been extensively investigated. Zheng et al. demonstrated a significant association between mid-to-late gestational hyperglycemia and neonatal metabolism involving arginine, proline, and the urea cycle (34). The pathophysiological mechanisms of HDP, such as placental insufficiency, oxidative stress, inflammation, and disruptions in fetal nutrient transport, lead to fetal metabolism disturbance (35–37). The levels of neonatal amino acids and acylcarnitine can largely reflect the status of fetal metabolism. Moreover, a recent study has revealed a significant relationship between changes in neonatal amino acid and acylcarnitine profiles and HDP (38). However, the effects of different types of HDP on these profiles require further investigation.

Therefore, the aim of this study was to leverage a relatively large study sample to investigate the effects of in utero HDP on neonatal amino acid and acylcarnitine levels and examine variations across different types of HDP. Blood metabolites were evaluated to elucidate how gestational hypertension influences the metabolic status of newborns.

## 2 Materials and methods

### 2.1 Study design and population

This retrospective case–control study included 1,228 singleton pregnant women with singleton pregnancies diagnosed with HDP and 1,228 women with normal singleton pregnancies, all of whom underwent NBS for amino acids and acylcarnitines. Normal pregnancy was defined as pregnancy without prenatal complications, such as HDP, intrahepatic cholestasis, and gestational diabetes mellitus. The participant inclusion criteria were as follows: (I) singleton pregnant women; (II) NBS for amino acids and acylcarnitines; and (III) delivery in Fujian Provincial Maternity and Child Hospital. The exclusion criteria were as follows: (I) multiple pregnancies; (II) stillbirth, neonatal death, fetal congenital abnormalities, and genetic metabolic diseases; (III) liver, heart, or renal disease; (IV) other perinatal complications, such as intrahepatic cholestasis during pregnancy, gestational diabetes mellitus, and heart disease; and (V) no NBS for amino acids and acylcarnitines.

**Figure 1:**
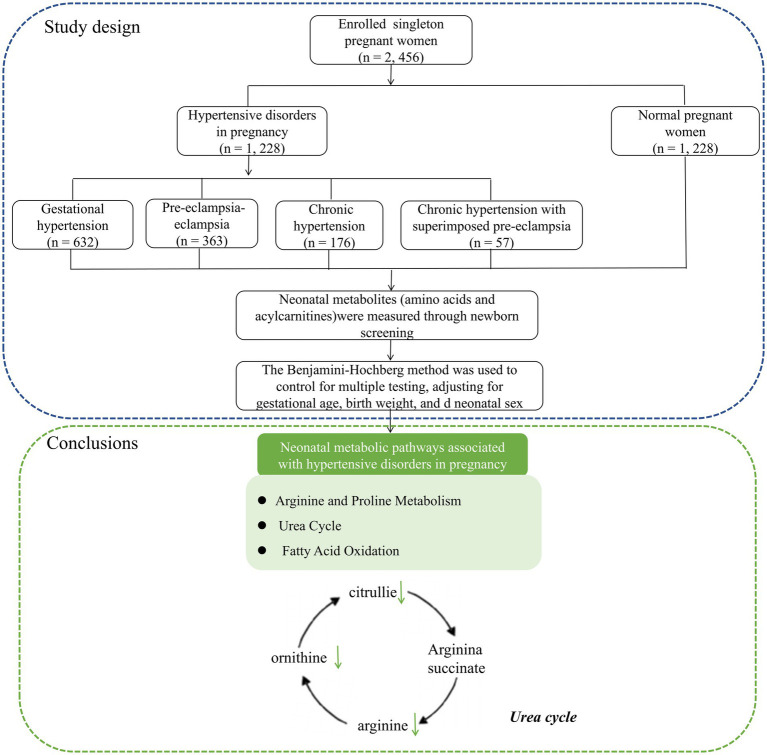
The workflow of the study design and neonatal metabolic pathways associated with hypertensive disorders in pregnancy.

Data were collected from the Fujian Maternity and Child Health Hospital between January 2021 and December 2023. Figure 1 illustrates the workflow of the study design. This study was conducted in accordance with the guidelines of the Declaration of Helsinki and approved by the Ethics Review Committee of Fujian Provincial Maternity and Child Hospital (approval no. 2024KY150), Fuzhou, China.

### 2.2 Blood pressure measurements and definition of hypertensive disorders in pregnancy

Maternal blood pressure (BP) was measured by trained nurses at every prenatal check-up, specifically at 6–12 weeks, 20–24 weeks, and 28–34 weeks of gestation, following the Chinese expert consensus on BP management during pregnancy. Before measurements, pregnant women were instructed to avoid smoking, eating, consuming alcohol or caffeinated drinks, and engaging in physical exercise for more than 30 min. They were also instructed to rest for at least 5 min in a comfortable environment. BP was taken from the right brachial artery in a sitting position using automated digital BP monitors, with the elbow positioned at heart level. The measurements were conducted twice, with a minimum interval of 2 min between each measurement. The average values of the two measurements were recorded. In cases where systolic blood pressure (SBP) was ≥ 160 mmHg and/or diastolic blood pressure (DBP) was ≥ 110 mmHg, BP was remeasured within 15 min. In cases where SBP was between 140 mmHg and 160 mmHg and/or DBP was between 90 mmHg and 110 mmHg, BP was remeasured within 4–6 h (39). Maternal age, gravida, para, and delivery-related clinical data, including gestational age at delivery, birth weight, sex, and Apgar scores, were also recorded.

HDP was divided into four categories (40): (a) GH, (b) PE, (c) CH, and (d) CH + PE. GH was defined as hypertension (mean SBP ≥ 140 mmHg or DBP ≥ 90 mmHg) first occurring after 20 weeks of pregnancy without proteinuria and resolving within 12 weeks postpartum. PE was defined as hypertension (mean SBP ≥ 140 mmHg and/or DBP ≥ 90 mmHg) occurring after 20 weeks of gestation, accompanied by proteinuria (random urine protein ≥ 1 + or urine protein quantification ≥ 300 mg/24 h) or without proteinuria but with damage to one organ or system. CH was defined as hypertension (≥140/90 mmHg) occurring prior to pregnancy or before 20 weeks of gestation. CH + PE was defined as chronic hypertension associated with PE.

In addition, the diagnosis of FGR is based on the Chinese Expert Consensus on Fetal Growth Restriction (An Update on the 2019 Version) (41).

### 2.3 NBS and biochemical analysis

The workflow of NBS was conducted according to our previously described procedures (42, 43). Briefly, blood spot samples were collected from the heel of newborns, air-dried at room temperature (25°C), and transported to the NBS center in Fujian province using a cold-chain transportation system. The concentrations of amino acids and acylcarnitines in the dried blood spot samples were quantified using a tandem mass spectrometry (MS/MS) system (ACQUITY TQD, Waters, USA) with a NeoBase™ MS/MS Kit (PerkinElmer, Turku, Finland). Briefly, dried blood spot and quality control samples (3.2 mm) were punched into 96-well plates. Then, 100 μL of the internal standard-containing routine working solution was added to each well, followed by incubation for 45 min in a shaking incubator (MB100-2A; Thermo) at 45°C and 750 rpm. The reaction was then terminated, and the contents of each well (75 μL) were transferred to a deep-well plate for a 2-h incubation. Flow injection analysis–MS/MS was used to measure internal standards and sample products using multiple reaction monitoring. Parameters of the liquid tandem mass spectrometer are shown in Supplementary Table S1. The amino acid profile included alanine, arginine, citrulline, glycine, leucine\isoleucine\hydroxyproline (Leu\Ile\Pro-OH), methionine, ornithine, phenylalanine, proline, valine, and tyrosine. The acylcarnitine profile included free carnitine (C0), acetylcarnitine (C2), propionylcarnitine (C3), malonylcarnitine + 3-hydroxybutyrylcarnitine (C3DC + C4OH), butyrylcarnitine + isobutyrylcarnitine (C4), methylmalonyl + 3-hydroxy-isovalerylcarnitine (C4DC + C5OH), isovalerylcarnitine + methylbutyrylcarnitine (C5), tiglylcarnitine (C5:1), glutarylcarnitine + 3-hydroxyhexanoylcarnitine (C5DC + C6OH), hexanoylcarnitine (C6), methylglutarylcarnitine (C6DC), octanoylcarnitine (C8), octenoylcarnitine (C8:1), decanoylcarnitine (C10), decenoylcarnitine (C10:1), decadienoylcarnitine (C10:2), dodecanoylcarnitine (C12), dodecenoylcarnitine (C12:1), tetradecanoylcarnitine (C14), tetradecenoylcarnitine (C14:1), tetradecadienoylcarnitine (C14:2), 3-hydroxy-tetradecanoylcarnitine (C14OH), palmitoylcarnitine (C16), palmitoleylcarnitine (C16:1), 3-hydroxy-hexadecanoylcarnitine (C16OH), 3-hydroxy-hexadecenoylcarnitine (C16:1OH), stearoylcarnitine (C18), oleoylcarnitine (C18:1), 3-hydroxy-octadecenoylcarnitine (C18:1OH), linoleoylcarnitine (C18:2), and 3-hydroxy-octadecanoylcarnitine (C18OH).

### 2.4 Statistical analyses

Statistical analyses were performed using R version 4.3.1^1^ and IBM SPSS Statistics for Windows (version 26.0; IBM Corp., Armonk, NY, USA). The ropls package was employed to perform partial least squares discriminant analysis (PLS-DA) to demonstrate the overall pattern of the data. The effects of grouping, gestational age, birth weight, and infant sex on metabolite distribution were evaluated using permutation-based multivariate analysis of variance (PERMANOVA). Volcano plots and heatmaps of differential metabolites were constructed using the ggplot2 and ComplexHeatmap packages, respectively. Categorical data are presented as frequency (*n*) and percentage (%), and intergroup comparisons were made using the chi-square test or Fisher’s exact test. Normally distributed continuous variables are expressed as mean ± standard deviation, independent samples *t*-tests were used for comparison between two groups, and one-way analysis of variance was used for comparisons among multiple groups. Non-normally distributed variables are presented as median and interquartile range, and the Kruskal–Wallis test was employed for multiple groups. Pairwise *post-hoc* tests to identify specific group differences were conducted using the Tukey–Kramer method. A *p* < 0.05 was considered statistically significant. The Benjamini–Hochberg method was employed to adjust for multiple testing, with the false discovery rate threshold set at 0.05.

## 3 Results

### 3.1 Basic clinical characteristics of the case–control study

A total of 2,456 women with singleton pregnancies and their newborns were enrolled in this study, comprising 1,228 women with HDP and 1,228 women with normal pregnancies. Among the HDP cases, there were 632 cases of GH, 363 cases of PE, 176 cases of CH, and 57 cases of CH + PE. The maternal age, gravida, para, gestational age at delivery, birth weight, and infant sex are presented in Table 1.

**Table 1:** Comparisons of neonatal basic clinical data between mothers with hypertensive disorders in pregnancy and those with normal pregnancy.

Variable	Gestational hypertension group (*N* = 632) Median or *n* (%)	Pre-eclampsia group (*N* = 363) Median or *n* (%)	Chronic hypertension group (*N* = 176) Median or *n* (%)	Chronic hypertension superimposed pre-eclampsia group (*N* = 57) Median or *n* (%)	Normal group (*N* = 1,228) Median or *n* (%)	*Z* or *χ*^2^-value	*p*
Gestational age at delivery (week)	38 (38, 39)	38 (37, 39)	38 (37.5, 39)	38 (37, 39)	39.5 (39, 40.2)	425.7	<0.001
Birth weight (g)	3,070 (2,820, 3,385)	3,145 (2,885, 3,375)	3,170 (2,925, 3,402)	3,085 (2,707, 3,415)	3,272 (3,065, 3,540)	119.8	<0.001
Gender (male)	329 (52.06)	176 (48.48)	93 (52.84)	28 (49.12)	661 (53.83)	3.523	0.474

No significant differences in maternal age, gravida, para, and infant sex were observed between the HDP and normal pregnancy groups (*p* > 0.05). However, significant differences in gestational age at delivery and birth weight were observed between the two groups (*p* < 0.001). Newborns born to mothers with GH, PE, CH, or CH + PE exhibited lower gestational ages and birth weights than those born to mothers with normal pregnancies. Furthermore, by comparing the birth weights of different HDP subgroups, we found that the birth weight in the PE group was lower than that in the GH group (*p* = 0.009), whereas the differences in other subgroups were not statistically significant (*p* > 0.05). Additionally, no significant differences in gestational age were found among the various HDP subgroups (*p* > 0.05). The effects of gestational age, birth weight, and infant sex on metabolite distribution were evaluated using PERMANOVA. The results showed significant differences between the HDP and normal pregnancy groups (Supplementary Table S2).

### 3.2 Comparisons of amino acid levels in the peripheral blood of newborns born to mothers with HDP and those born to mothers with normal pregnancies

**Figure 2:**
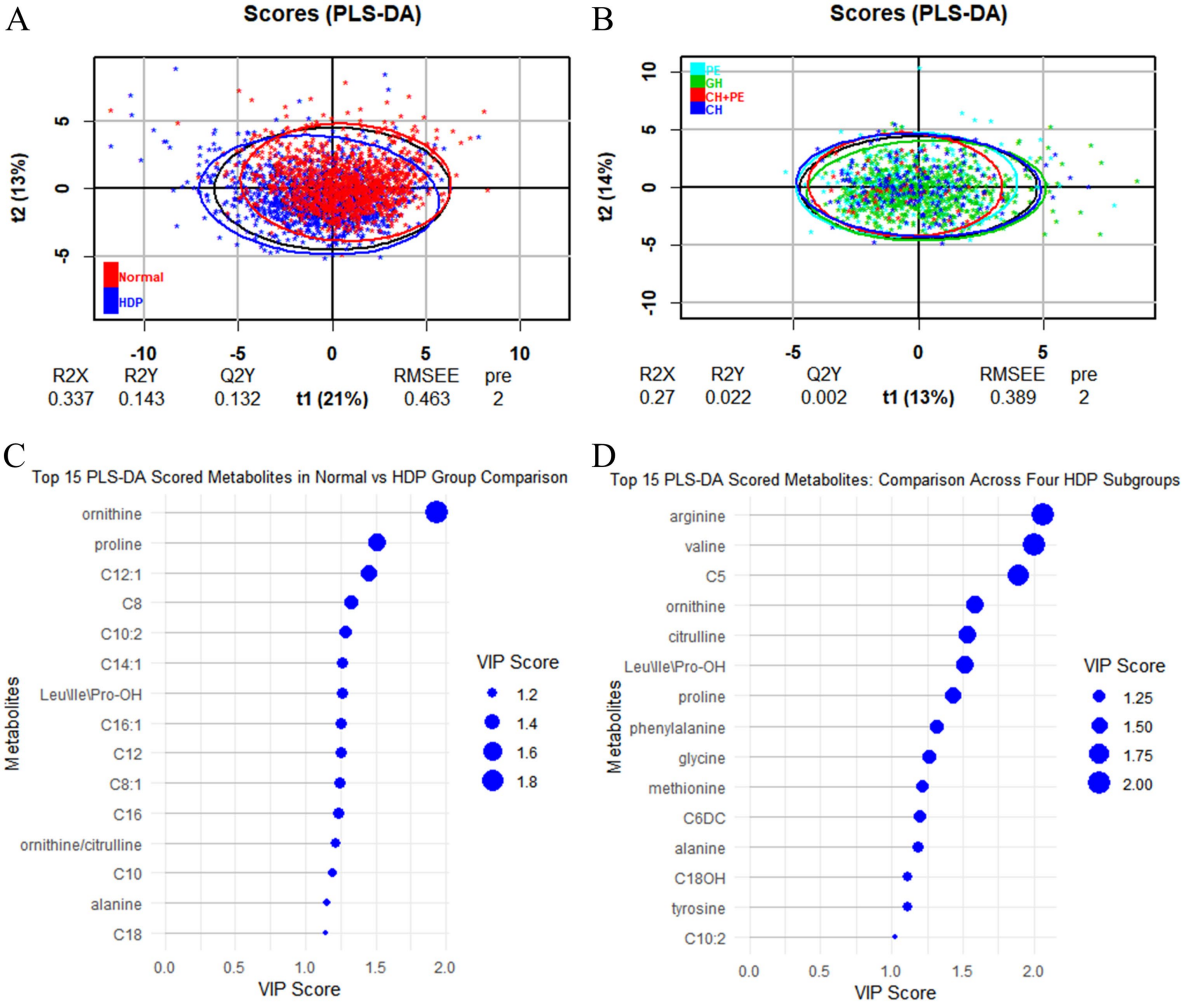
PLS-DA analysis between the HDP and normal pregnancy groups and among the four HDP subgroups. **(A)** HDP vs. Normal pregnancy. **(B)** The four HDP subgroups. **(C)** The top 15 VIP scores of the metabolites in HDP vs. Normal pregnancy group comparison. **(D)** The top 15 VIP scores of the metabolites in the four HDP subgroups comparison. HDP, hypertensive disorders in pregnancy; GH, gestational hypertension; PE, pre-eclampsia; CH, chronic hypertension; CH + PE, chronic hypertension superimposed pre-eclampsia; VIP, variable importance in projection; PLS-DA, partial least squares discriminant analysis; Leu\Ile\Pro-OH, leucine\isoleucine\hydroxyproline; C5, sovalerylcarnitine + methylbutyrylcarnitine; C6DC, methylglutarylcarnitine; C8, octanoylcarnitine; C8:1, octenoylcarnitine; C10, decanoylcarnitine; C10:2, decadienoylcarnitine; C12, dodecanoylcarnitine; C12:1, dodecenoylcarnitine; C14:1, tetradecenoylcarnitine; C16, palmitoylcarnitine; C16:1, palmitoleylcarnitine; C18, stearoylcarnitine.

**Figure 3:**
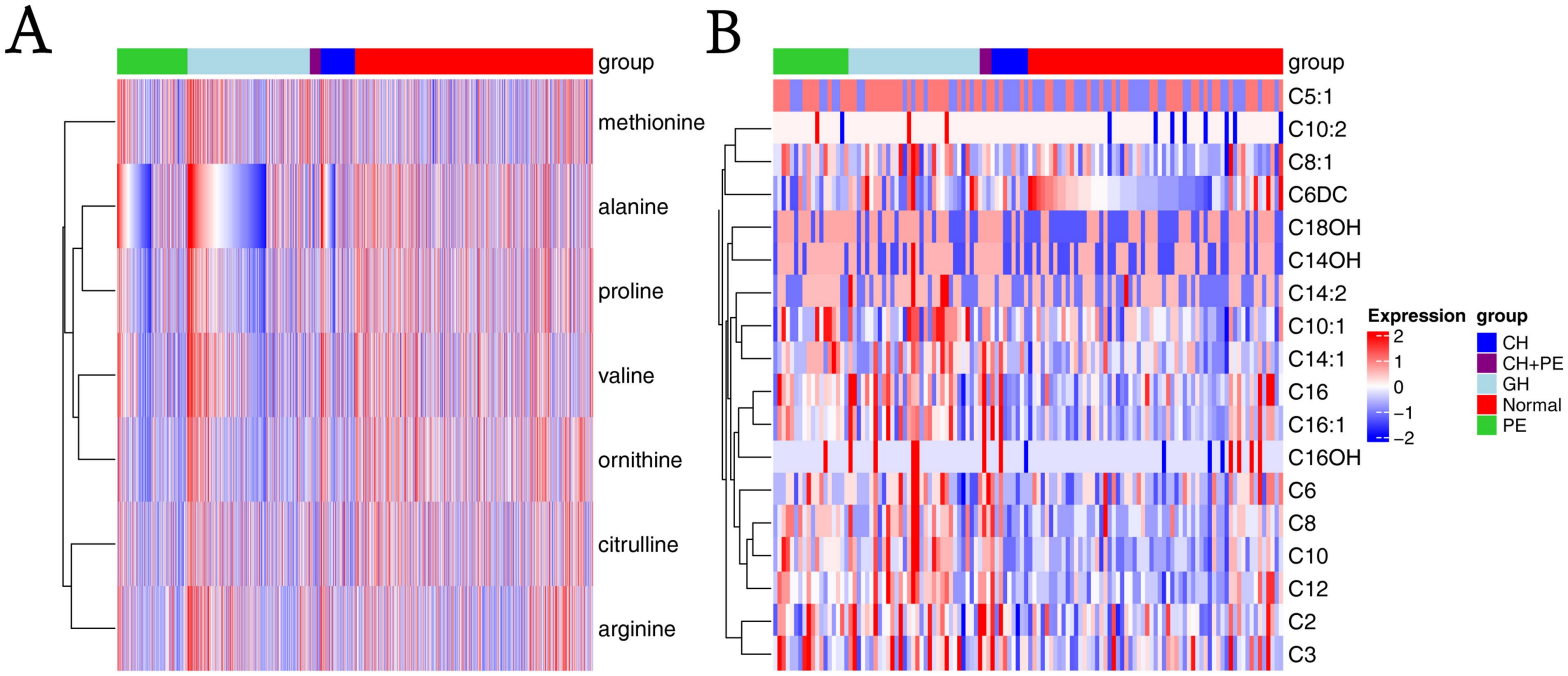
Heatmaps based on metabolite groups. **(A)** Amino acids; **(B)** Carnitines. CH, chronic hypertension; CH + PE, chronic hypertension with superimposed pre-eclampsia; GH, gestational hypertension; PE, pre-eclampsia-eclampsia; C2, acetylcarnitine; C3, propionylcarnitine; C5:1:tiglylcarnitine; C6, hexanoylcarnitine; C6DC, methylglutarylcarnitine; C8, octanoylcarnitine; C8:1, octenoylcarnitine; C10, decanoylcarnitine; C10:1, decenoylcarnitine; C10:2, decadienoylcarnitine; C12, dodecanoylcarnitine; C14:1, tetradecenoylcarnitine; C14:2, tetradecadienoylcarnitine; C14OH, 3-hydroxy-tetradecanoylcarnitine; C16, palmitoylcarnitine; C16:1, palmitoleylcarnitine; C16OH, 3-hydroxy-hexadecanoylcarnitine; C18OH, 3-hydroxy-octadecanoylcarnitine.

All samples from the HDP and normal pregnancy groups were analyzed by PLS-DA. No clear separation between the HDP and normal pregnancy groups or among the four HDP subgroups was observed in the PLS-DA plot. The top 15 variable importance in projection scores of the metabolites are presented in Figure 2. We further evaluated the impact of HDP on neonatal amino acid metabolism, the levels of 11 specific amino acids, and the ratio of tyrosine/phenylalanine and ornithine/citrulline. Except for arginine and phenylalanine, significant differences were observed in the levels of the nine remaining amino acids, as well as the ratio of tyrosine/phenylalanine and ornithine/citrulline between infants born to mothers with HDP and those born to mothers with normal pregnancies (*p* < 0.05) (Table 2). The overall levels of alanine, citrulline, ornithine, Leu\Ile\Pro-OH, proline, tyrosine, and valine in newborns in the HDP group were lower than those in newborns in the normal pregnancy group, whereas the overall level of glycine and methionine in newborns in the HDP group was higher. The comparisons of multiple groups among the normal pregnancy group and HDP subgroups, including GH, PE, CH, and CH + PE were conducted. Except for glycine and phenylalanine, significant differences were observed in the levels of the nine remaining amino acids and the ratios of tyrosine/phenylalanine and ornithine/citrulline among the normal pregnancy group and the HDP subgroups (*p* < 0.001) (Table 3; Figure 3A).

**Table 2:** Comparisons of amino acid and acylcarnitine levels in peripheral blood of newborns between mothers with hypertensive disorders in pregnancy and those with normal pregnancy.

Amino acid or Acylcarnitine	HDP group (*N* = 1,228) Media (P25, P75)	Normal group (*N* = 1,228) Media (P25, P75)	*Z*-value	*p*	FDR
Alanine	298.8 (248.4, 355.1)	324.9 (279.7, 379.8)	8.397	< 0.001	< 0.001
Arginine	8.3 (5.0, 14.5)	8.8 (5.1, 14.7)	1.266	0.205	0.236
Citrulline	10.9 (9.0, 13.3)	11.7 (9.8, 14.3)	6.012	< 0.001	< 0.001
Glycine	403.6 (354.0, 461.6)	395.6 (350.0, 455.8)	2.261	0.024	0.033
Leu\Ile\Pro-OH	152.4 (129.1, 177.6)	164.9 (142.3, 189.6)	8.605	< 0.001	< 0.001
Methionine	23.8 (20.3, 27.7)	22.8 (19.7, 26.5)	4.478	< 0.001	< 0.001
Ornithine	102.9 (84.4, 124.0)	121.3 (100.6, 147.7)	13.646	< 0.001	< 0.001
Phenylalanine	50.6 (44.3,57.9)	50.5 (44.0, 57.9)	0.452	0.651	0.680
Proline	163.9 (141.3, 192.0)	181.3 (156.0, 211.2)	10.314	< 0.001	< 0.001
Tyrosine	89.8 (71.8, 114.4)	94.4 (74.3, 116.7)	3.065	0.002	0.003
Valine	119.3 (101.3,142.6)	128.2 (110.6, 147.1)	6.407	< 0.001	< 0.001
Tyrosine/phenylalanine ratio	1.72 (1.40. 2.21)	1.85 (1.45, 2.35)	3.867	< 0.001	< 0.001
Ornithine/citrulline ratio	9.51 (7.83, 11.43)	10.35 (8.57,12.67)	7.637	< 0.001	< 0.001
C0	22.07 (18.41, 27.65)	22.91 (19.16, 27.52)	1.987	0.047	0.058
C2	18.93 (15.36, 23.34)	17.54 (14.41, 21.23)	6.327	< 0.001	< 0.001
C3	1.56 (1.22, 1.98)	1.43 (1.14, 1.85)	5.106	< 0.001	< 0.001
C3DC + C4OH	0.08 (0.07, 0.11)	0.07 (0.06, 0.10)	6.750	< 0.001	< 0.001
C4	0.20 (0.17, 0.23)	0.20 (0.17, 0.23)	0.544	0.586	0.626
C4DC + C5OH	0.17 (0.14, 0.20)	0.18 (0.15, 0.21)	2.793	0.005	0.008
C5	0.10 (0.08, 0.12)	0.10 (0.08, 0.12)	0.206	0.837	0.855
C5:1	0.05 (0.00, 0.10)	0.00 (0.00, 0.01)	2.277	0.023	0.032
C5DC + C6OH	0.09 (0.08, 0.11)	0.09 (0.07, 0.11)	5.940	< 0.001	< 0.001
C6	0.04 (0.03, 0.05)	0.03 (0.03, 0.04)	6.040	< 0.001	< 0.001
C6DC	0.12 (0.09, 0.15)	0.12 (0.10, 0.16)	4.022	< 0.001	< 0.001
C8	0.04 (0.03, 0.05)	0.04 (0.03, 0.05)	9.477	< 0.001	< 0.001
C8:1	0.11 (0.09, 0.14)	0.10 (0.08, 0.12)	7.849	< 0.001	< 0.001
C10	0.05 (0.04, 0.07)	0.05 (0.04, 0.06)	7.940	< 0.001	< 0.001
C10:1	0.06 (0.05, 0.08)	0.06 (0.04, 0.07)	3.502	< 0.001	< 0.001
C10:2	0.01 (0.01, 0.01)	0.01 (0.01, 0.01)	8.259	< 0.001	< 0.001
C12	0.06 (0.05, 0.07)	0.05 (0.04, 0.07)	8.579	< 0.001	< 0.001
C12:1	0.05 (0.03, 0.07)	0.05 (0.03, 0.07)	0.166	0.868	0.868
C14	0.16 (0.13, 0.20)	0.16 (0.13, 0.19)	1.715	0.086	0.104
C14:1	0.07 (0.06, 0.09)	0.07 (0.05, 0.09)	4.135	< 0.001	< 0.001
C14:2	0.02 (0.01, 0.02)	0.02 (0.01, 0.02)	5.665	< 0.001	< 0.001
C14OH	0.01 (0.00, 0.01)	0.01 (0.00, 0.01)	6.737	< 0.001	< 0.001
C16	2.34 (1.76,2.98)	2.18 (1.70, 2.77)	3.627	< 0.001	< 0.001
C16:1	0.11 (0.08,0.15)	0.10 (0.07, 0.13)	5.702	< 0.001	< 0.001
C16OH	0.01 (0.01, 0.01)	0.01 (0.01, 0.01)	2.434	0.015	0.022
C16:1OH	0.03 (0.02, 0.04)	0.03 (0.03, 0.04)	0.772	0.440	0.481
C18	0.73 (0.58, 0.90)	0.70 (0.57, 0.87)	2.116	0.034	0.045
C18:1	1.31 (1.08, 1.55)	1.28 (1.07, 1.49)	2.213	0.027	0.036
C18:1OH	0.01 (0.01, 0.02)	0.01 (0.01, 0.02)	2.042	0.041	0.052
C18:2	0.24 (0.18, 0.31)	0.24 (0.19, 0.30)	0.844	0.399	0.446
C18OH	0.01 (0.00, 0.01)	0.01 (0.00, 0.01)	4.878	< 0.001	< 0.001

HDP, hypertensive disorders in pregnancy; FDR, false discovery rate; Leu\Ile\Pro-OH, leucine\isoleucine\hydroxyproline; C0, free carnitine; C2, acetylcarnitine; C3, propionylcarnitine; C3DC + C4OH, malonylcarnitine + 3-hydroxybutyrylcarnitine; C4, butyrylcarnitine + isobutyrylcarnitine; C4DC + C5OH, methylmalonyl + 3-hydroxy-isovalerylcarnitine; C5, sovalerylcarnitine + methylbutyrylcarnitine; C5:1:tiglylcarnitine; C5DC + C6OH, glutarylcarnitine + 3- hydroxyhexanoylcarnitine; C6, hexanoylcarnitine; C6DC, methylglutarylcarnitine; C8, octanoylcarnitine; C8:1, octenoylcarnitine; C10, decanoylcarnitine; C10:1, decenoylcarnitine; C10:2, decadienoylcarnitine; C12, dodecanoylcarnitine; C12:1:dodecenoylcarnitine; C14, ltetradecanoylcarnitine; C14:1, tetradecenoylcarnitine; C14:2, tetradecadienoylcarnitine; C14OH, 3-hydroxy-tetradecanoylcarnitine; C16, palmitoylcarnitine; C16:1, palmitoleylcarnitine; C16OH, 3-hydroxy-hexadecanoylcarnitine; C16:1OH, 3-hydroxy-hexadecenoylcarnitine; C18, stearoylcarnitine; C18:1, oleoylcarnitine; C18:1OH, 3-hydroxy-octadecenoylcarnitine; C18:2, linoleoylcarnitine; C18OH, 3-hydroxy-octadecanoylcarnitine.

**Table 3:** Comparisons of amino acid levels in peripheral blood of newborns born to mothers with the normal pregnancy and the subgroups of the hypertensive disorders in pregnancy.

Amino acid	Gestational hypertension group (*N* = 632) Media (P25, P75)	Pre-eclampsia group (*N* = 363) Media (P25, P75)	Chronic hypertension group (*N* = 176) Media (P25, P75)	Chronic hypertension superimposed pre-eclampsia group (*N* = 57) Media (P25, P75)	Normal group (*N* = 1,228) Media (P25, P75)	*Z*-value	*p*	FDR
Alanine	303.6 (248.9, 359.2)	291.8 (247.4, 347.3)	299.6 (250.1, 359.2)	286.1 (241.2, 350.8)	324.8 (279.3, 379.6)	71.71	<0.001	<0.001
Arginine	9.285 (5.675, 15.96)	7.200 (4.525, 12.02)	8.605 (4.525, 14.35)	5.770 (3.120, 12.99)	8.750 (5.090, 14.85)	34.46	<0.001	<0.001
Citrulline	11.34 (9.400, 13.67)	10.40 (8.770, 12.66)	10.46 (8.630, 13.34)	10.21 (8.700, 13.50)	11.69 (9.780, 14.33)	55.02	<0.001	<0.001
Glycine	404.7 (352.57, 464.4)	402.4 (350.2, 457.5)	403.4 (362.8, 466.1)	405.4 (360.4, 444.6)	395.6 (349.9, 455.0)	5.480	0.250	0.287
Leu\Ile\Pro-OH	153.1 (129.6, 179.9)	146.4 (127.0, 169.8)	157.0 (135.6, 185.2)	153.4 (122.5, 171.4)	164.9 (142.3, 189.4)	84.92	<0.001	<0.001
Methionine	24.24 (20.82, 28.24)	23.52 (19.98, 27.14)	23.54 (20.10, 27.60)	22.74 (19.71, 25.95)	22.74 (19.68, 26.47)	30.22	<0.001	<0.001
Ornithine	104.1 (86.72, 129.4)	99.65 (81.94, 117.3)	105.1 (85.65, 127.4)	101.2 (78.40, 118.0)	121.7 (100.8, 147.9)	204.8	<0.001	<0.001
Phenylalanine	50.55 (44.44, 57.58)	50.34 (43.72, 57.28)	51.43 (44.23, 59.46)	51.92 (46.14, 59.95)	50.44 (43.96, 57.79)	3.733	0.465	0.509
Proline	166.9 (142.7, 198.3)	159.2 (138.5, 185.6)	166.8 (141.1, 196.7)	159.2 (134.4, 180.5)	181.0 (155.9, 211.0)	113.4	<0.001	<0.001
Tyrosine	91.83 (73.06, 115.4)	85.67 (67.68, 109.5)	91.68 (76.14, 121.7)	88.64 (74.30, 106.4)	94.54 (74.32, 116.7)	20.73	<0.001	<0.001
Valine	123.9 (103.5, 146.2)	114.9 (98.72, 133.5)	120.9 (99.75, 145.8)	116.1 (101.1, 132.0)	128.1 (110.5, 146.7)	62.34	<0.001	<0.001
Tyrosine/phenylalanine ratio	1.8 (1.4, 2.3)	1.7 (1.4, 2.1)	1.7 (1.4, 2.3)	1.7 (1.4, 2.0)	1.9 (1.5, 2.4)	23.55	<0.001	<0.001
Ornithine/citrulline ratio	9.3 (7.7, 11.4)	9.5 (7.8, 11.4)	9.8 (8.6, 11.9)	9.8 (7.2, 11.5)	10.3 (8.6, 12.7)	63.10	<0.001	<0.001

FDR, false discovery rate; Leu\Ile\Pro-OH, leucine\isoleucine\hydroxyproline.

**Figure 4:**
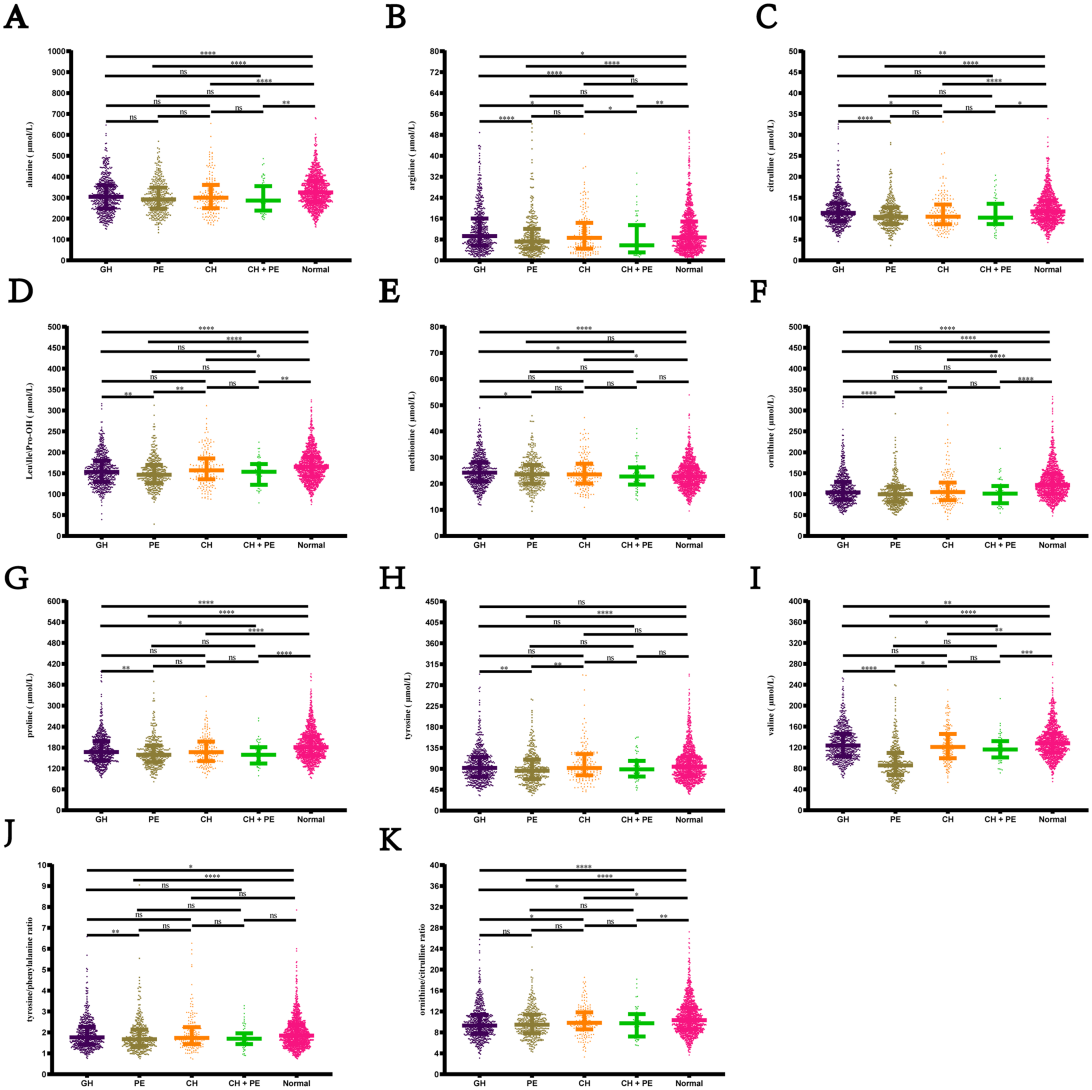
Pairwise comparisons of the levels of amino acids among the subgroups within the hypertensive disorders in pregnancy and normal pregnancy groups. **(A)** Alanine; **(B)** Arginine; **(C)** citrulline; **(D)** Leu\Ile\Pro-OH; **(E)** Methionine; **(F)** Ornithine; **(G)** Proline; **(H)** Tyrosine; **(I)** Citrulline; **(J)** Tyrosine/phenylalanine ratio; **(K)** Ornithine/citrulline ratio. Data are presented as median with interquartile range. *p < 0.05, **p < 0.01, ***p < 0.001, ****p < 0.0001. Leu\Ile\Pro-OH, leucine\isoleucine\hydroxyproline; GH, gestational hypertension; PE, pre-eclampsia; CH, chronic hypertension; CH + PE, chronic hypertension superimposed pre-eclampsia.

Pairwise comparisons among the subgroups within the HDP and normal pregnancy groups (Figure 4) revealed that newborns born to mothers with GH, PE, CH, and CH + PE exhibited lower levels of alanine, citrulline, ornithine, proline, and valine, as well as the ratio of ornithine/citrulline, than those born to mothers with normal pregnancies. Infants in the PE group had significantly lower levels of alanine, citrulline, Leu\Ile\Pro-OH, methionine, ornithine, proline, tyrosine, and valine than those in the GH group. Although amino acids levels showed a decreasing trend in the CH + PE group compared to the CH, no statistically significant differences were found between these groups, except for alanine. No significant differences were observed in the levels of the 11 amino acids between the PE and CH + PE groups.

**Figure 5:**
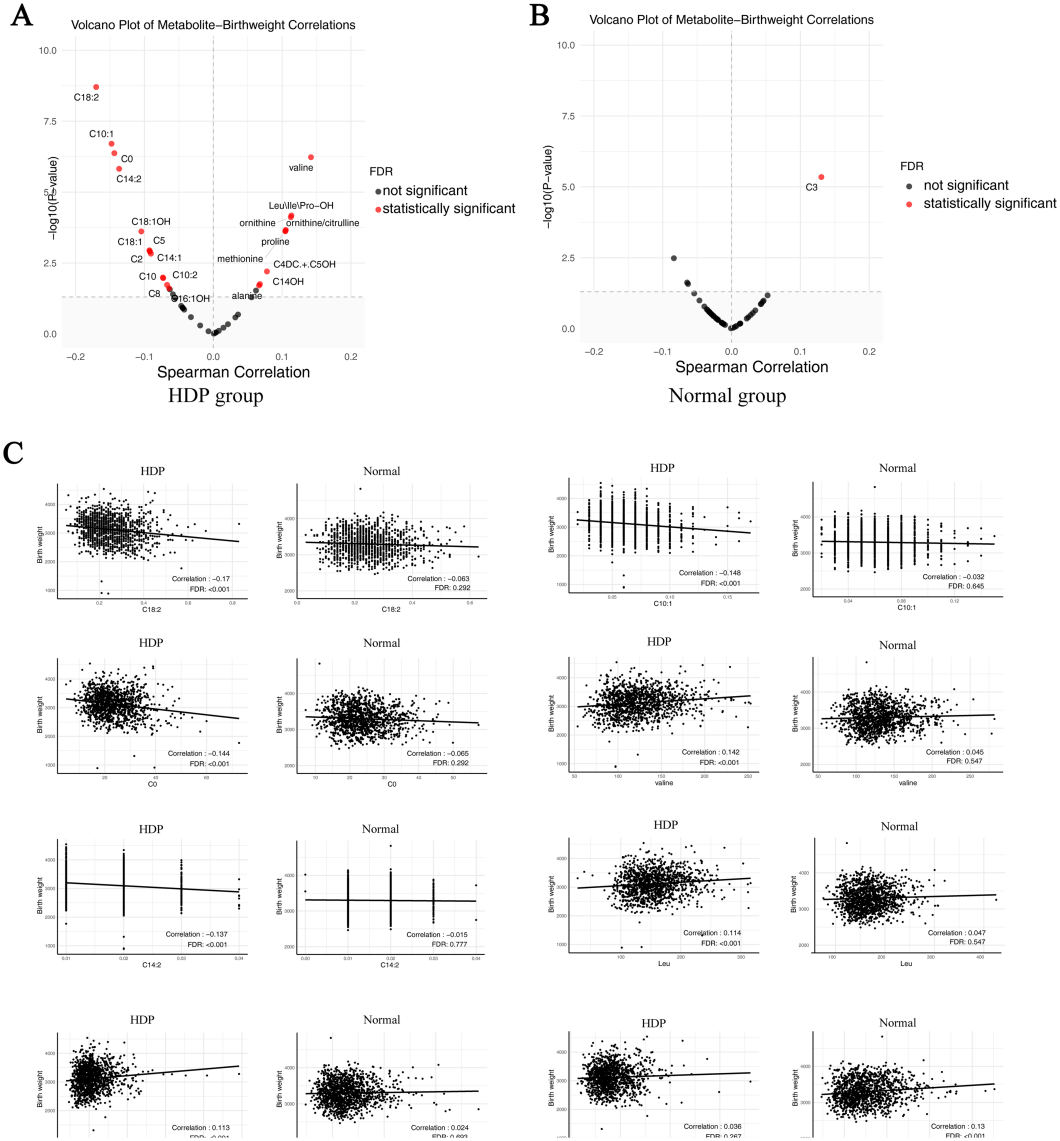
Correlation plot of metabolites with newborn birth weight in the HDP and normal pregnancy groups. **(A)** Correlation plot of metabolites with newborn birth weight in the HDP group. **(B)** Correlation plot of metabolites with newborn birth weight in the normal pregnancy group. **(C)** The scatter plot of top 8 pairwise correlations between metabolites and birth weight. HDP, hypertensive disorders in pregnancy; Leu\Ile\Pro-OH, leucine\isoleucine\hydroxyproline; C0, free carnitine; C2, acetylcarnitine; C3, propionylcarnitine; C4DC + C5OH, methylmalonyl + 3-hydroxy-isovalerylcarnitine; C5, sovalerylcarnitine + methylbutyrylcarnitine; C8, octanoylcarnitine; C10, decanoylcarnitine; C10:1, decenoylcarnitine; C10:2, decadienoylcarnitine; C14:1, tetradecenoylcarnitine; C14:2, tetradecadienoylcarnitine; C14OH, 3-hydroxy-tetradecanoylcarnitine; C16:1OH, 3-hydroxy-hexadecenoylcarnitine; C18:1, oleoylcarnitine; C18:1OH, 3-hydroxy-octadecenoylcarnitine; C18:2, linoleoylcarnitine.

We performed sex-stratified analyses between the HDP group and normal pregnancy group to explore sex-specific metabolite differences. Regardless of whether the infants were males or females, differences could be observed between the two groups in the overall alanine, citrulline, Leu/Ile/Pro-OH, methionine, ornithine, and valine levels, as well as in the ornithine/citrulline ratios (Supplementary Table S3). We also performed the amino acid and birth weight correlation analyses. The results indicated that birth weight was positively correlated with valine, Leu\Ile\Pro-OH, ornithine, methionine, and proline in the HDP group (*p* < 0.05) (Figure 5A), whereas no significant correlations were observed in the normal pregnancy group (Figure 5B). The scatter plot of the statistically significant correlations between certain amino acids and birth weight in the HDP group are presented in Figure 5C.

Gestational age and birth weight are considered to significantly influence amino acid metabolism in newborns (34, 44, 45). Thus, a linear regression analysis, adjusting for gestational age, birth weight, and neonatal sex, revealed significant associations between the HDP subgroups and amino acid levels (Table 4). After adjusting for multiple comparisons using the Benjamini–Hochberg method, ornithine and proline levels were significantly lower in the GH, PE, CH, and CH + PE groups than in the normal pregnancy group. Conversely, glycine and methionine levels were significantly elevated in infants born to mothers with GH, PE, and CH. Additionally, Leu\Ile\Pro-OH and tyrosine levels were significantly lower in the GH, PE, and CH + PE groups than in the normal pregnancy group.

**Table 4:** Associations between hypertensive disorders in pregnancy and amino acid levels in peripheral blood of newborns.

Amino acid	Pre-eclampsia group (*N* = 363)		Gestational hypertension group (*N* = 632)		Chronic hypertension superimposed pre-eclampsia group (*N* = 57)		Chronic hypertension group (*N* = 176)	
	β (95% CI)	FDR*	*β* (95% CI)	FDR*	*β* (95% CI)	FDR*	*β* (95% CI)	FDR*
Alanine	−11.935 (−21.738 to −2.133)	0.068	−8.308 (−16.175 to −0.442)	0.077	−18.501 (−39.070 to 2.068)	0.104	−6.716 (−19.139 to 5.707)	0.289
Arginine	−1.653 (−2.730 to −0.577)	0.011	0.830 (−0.034 to 1.694)	0.080	−2.310 (−4.569 to −0.051)	0.080	−0.592 (−1.956 to 0.773)	0.395
Citrulline	−0.980 (−1.455 to −0.505)	0.000	−0.162 (−0.543 to 0.220)	0.406	−0.870 (−1.868 to 0.127)	0.116	−0.707 (−1.309 to −0.104)	0.043
Glycine	10.990 (−0.282 to 22.263)	0.075	13.576 (4.529 to 22.622)	0.013	8.332 (−15.322 to 31.987)	0.490	17.880 (3.593 to 21.167)	0.028
Leu\Ile\Pro-OH	−13.007 (−17.822 to −8.192)	0.000	−8.361 (−12.225 to −4.497)	0.000	−13.811 (−23.915 to −3.708)	0.009	−3.017 (−9.119 to 3.086)	0.333
Methionine	1.267 (0.556 to 1.977)	0.001	1.967 (1.397 to 2.537)	0.000	0.484 (−1.001 to 1.974)	0.525	1.554 (0.654 to 2.454)	0.001
Ornithine	−17.139 (−22.078 to −12.200)	0.000	−10.393 (−14.357 to −6.430)	0.000	−19.614 (−29.978 to −9.250)	0.000	−11.138 (−17.398 to −4.878)	0.000
Phenylalanine	−0.368 (−1.741 to 1.006)	0.800	−0.104 (−1.206 to 0.998)	0.853	1.673 (−1.209 to 4.556)	0.511	1.088 (−0.653 to 2.829)	0.511
Proline	−14.047 (−19.638 to −8.455)	0.000	−7.886 (−12.373 to −3.399)	0.000	−20.491 (−32.224 (−8.758))	0.001	−10.148 (−17.235to − 3.062)	0.005
Tyrosine	−14.814 (−19.613 to −10.014)	0.000	−7.859 (−11.711 to −4.008)	0.000	−12.516 (−22.587 to −2.445)	0.020	−2.762 (−8.845 to 3.321)	0.374
Valine	−7.410 (−11.286 to −3.532)	0.001	0.321 (−2.791 to 3.432)	0.849	−9.639 (−17.775 to −1.504)	0.041	−2.099 (−7.013 to 2.815)	0.537
Tyrosine/phenylalanine ratio	−0.281 (−0.377 to −0.185)	0.000	−0.160 (−0.236 to −0.083)	0.000	−0.306 (−0.507 to −0.106)	0.003	−0.091 (−0.212 to 0.030)	0.143
Ornithine/citrulline ratio	−0.775 (−1.175 to −0.376)	0.000	−0.788 (−1.109 to −0.467)	0.000	−0.876 (−1.715 to 0.038)	0.054	−0.440 (−0.947 to 0.066)	0.089

FDR, false discovery rate; Leu\Ile\Pro-OH, leucine\isoleucine\hydroxyproline.

The models were adjusted for gestational age at delivery, birth weight, and neonatal sex. *FDR was calculated to account for multi- ple comparisons using the Benjamini-Hochberg method.

FGR, a commonly observed complication in HDP constitutes one of the significant causes of neonatal adverse outcomes. To explore the relationship between neonatal metabolism and FGR, we compared the amino acids and acylcarnitines in HDP groups with and without FGR. Adjusting for gestational age, birth weight, and neonatal sex, the overall levels of arginine, Leu\Ile\Pro-OH, methionine, ornithine, valine, and ornithine/citrulline ratio in newborns in the HDP group with FGR were lower than those in newborns in the HDP group without FGR (Supplementary Table S4).

### 3.3 Comparisons of acylcarnitine levels in the peripheral blood of newborns born to mothers with HDP and those born to mothers with normal pregnancies

To assess the effects of HDP on metabolic disturbances in acylcarnitine profiles, the levels of 31 amino acids were measured and compared between newborns of mothers with HDP and those of mothers with normal pregnancies. Except for C4, C5, C12:1, C14, C16:1OH, and C18:2, significant differences were observed in the levels of the remaining acylcarnitines between the two groups (*p* < 0.05) (Table 2). The results of the multiple group comparisons showed significant differences in the levels of 21 acylcarnitines among the HDP subgroups and normal pregnancy group (*p* < 0.05) (Table 5; Figure 3B).

**Table 5:** Comparisons of the level of acylcarnitines in peripheral blood of newborns between mothers with hypertensive disorders in pregnancy and those with normal pregnancy.

Acylcarnitine	Gestational hypertension group (*N* = 632) Mean (SD)/Median	Pre-eclampsia group (*N* = 363) Mean (SD)/Median	Chronic hypertension group (*N* = 176) Mean (SD)/Median	Chronic hypertension superimposed pre-eclampsia group (*N* = 57) Mean (SD)/Median	Normal group (*N* = 1,228) Mean (SD)/Media	*Z*-value	*p*	FDR
C0	22.24 (18.16, 27.54)	22.13 (18.46, 27.84)	21.53 (18.70, 27.61)	22.00 (19.32 29.22)	22.91 (19.13, 27.46)	4.333	0.331	0.370
C2	18.74 (15.42, 23.31)	19.03 (15.46, 23.00)	18.76 (14.82, 23.24)	20.54 (16.87, 25.33)	17.52 (14.39, 21.24)	43.43	<0.001	<0.001
C3	1.55 (1.20, 2.06)	1.57 (1.23, 1.96)	1.578 (1.25, 2.02)	1.54 (1.20, 1.89)	1.43 (1.14, 1.84)	27.29	<0.001	<0.001
C3DC + C4OH	0.08 (0.07, 0.11)	0.08 (0.06, 0.10)	0.08 (0.07, 0.110)	0.08 (0.07, 0.11)	0.07 (0.06, 0.10)	46.91	<0.001	<0.001
C4	0.20 (0.17, 0.23)	0.20 (0.16, 0.24)	0.20 (0.17, 0.23)	0.20 (0.16, 0.23)	0.20 (0.17, 0.23)	1.073	0.939	0.939
C4DC + C5OH	0.17 (0.15, 0.20)	0.17 (0.14, 0.20)	0.17 (0.15, 0.20)	0.16 (0.14, 0.21)	0.18 (0.15, 0.21)	10.59	0.026	0.036
C5	0.10 (0.08, 0.12)	0.10 (0.08, 0.13)	0.10 (0.08, 0.12)	0.10 (0.07, 0.12)	0.10 (0.08, 0.12)	5.775	0.222	0.275
C5:1	0.01 (0.00, 0.01)	0.00 (0.00, 0.10)	0.00 (0.00, 0.01)	0.00 (0.00, 0.01)	0.00 (0.00, 0.01)	10.03	0.040	0.066
C5DC + C6OH	0.09 (0.08, 0.11)	0.09 (0.08, 0.11)	0.09 (0.08, 0.11)	0.10 (0.08, 0.11)	0.09 (0.07, 0.10)	39.46	<0.001	<0.001
C6	0.04 (0.03, 0.05)	0.04 (0.03, 0.05)	0.04 (0.03, 0.045)	0.04 (0.03, 0.04)	0.03 (0.03, 0.04)	39.35	<0.001	<0.001
C6DC	0.12 (0.09, 0.16)	0.11 (0.09, 0.14)	0.12 (0.09, 0.155)	0.11 (0.08, 0.13)	0.12 (0.10, 0.16)	24.54	<0.001	<0.001
C8	0.04 (0.03, 0.05)	0.04 (0.03, 0.05)	0.04 (0.03, 0.05)	0.04 (0.03, 0.05)	0.04 (0.03, 0.05)	98.47	<0.001	<0.001
C8:1	0.11 (0.09, 0.14)	0.11 (0.09, 0.14)	0.10 (0.08, 0.13)	0.10 (0.09, 0.13)	0.10 (0.08, 0.12)	71.63	<0.001	<0.001
C10	0.06 (0.04, 0.07)	0.05 (0.04, 0.07)	0.05 (0.04, 0.07)	0.05 (0.04, 0.06)	0.05 (0.04, 0.06)	67.76	<0.001	<0.001
C10:1	0.06 (0.05, 0.07)	0.06 (0.05, 0.08)	0.06 (0.045, 0.08)	0.06 (0.05, 0.08)	0.06 (0.04, 0.07)	14.57	0.006	0.008
C10:2	0.01 (0.01, 0.01)	0.01 (0.01, 0.01)	0.01 (0.01, 0.01)	0.01 (0.01, 0.01)	0.01 (0.01, 0.01)	75.64	<0.001	<0.001
C12	0.06 (0.05, 0.08)	0.06 (0.05, 0.07)	0.06 (0.05, 0.07)	0.06 (0.05, 0.07)	0.05 (0.04, 0.07)	76.14	<0.001	<0.001
C12:1	0.04 (0.03, 0.06)	0.04 (0.03, 0.07)	0.05 (0.03, 0.07)	0.05 (0.04, 0.07)	0.05 (0.03, 0.07)	5.600	0.230	0.278
C14	0.16 (0.13, 0.20)	0.16 (0.13, 0.20)	0.16 (0.13, 0.20)	0.16 (0.13, 0.20)	0.16 (0.13, 0.19)	2.949	0.566	0.604
C14:1	0.07 (0.06, 0.09)	0.07 (0.06, 0.09)	0.07 (0.06, 0.09)	0.08 (0.06, 0.09)	0.07 (0.05, 0.09)	23.15	<0.001	<0.001
C14:2	0.02 (0.01, 0.02)	0.02 (0.01, 0.02)	0.02 (0.01, 0.02)	0.02 (0.01, 0.02)	0.02 (0.01, 0.02)	33.63	<0.001	<0.001
C14OH	0.01 (0.00, 0.01)	0.01 (0.01, 0.01)	0.01 (0.00, 0.01)	0.01 (0.01, 0.01)	0.01 (0.00, 0.01)	43.48	<0.001	<0.001
C16	2.37 (1.81, 3.01)	2.28 (1.69, 2.92)	2.305 (1.73, 3.00)	2.41 (1.90, 2.98)	2.18 (1.70, 2.78)	16.64	0.002	0.003
C16:1	0.11 (0.08, 0.15)	0.11 (0.08, 0.14)	0.11 (0.07, 0.15)	0.10 (0.08, 0.16)	0.10 (0.07, 0.13)	33.36	<0.001	<0.001
C16OH	0.03 (0.02, 0.04)	0.03 (0.02, 0.04)	0.03 (0.02, 0.04)	0.03 (0.02, 0.04)	0.03 (0.03, 0.04)	10.83	0.026	0.036
C16:1OH	0.01 (0.01, 0.01)	0.01 (0.01, 0.01)	0.01 (0.01, 0.01)	0.01 (0.01, 0.01)	0.01 (0.01, 0.01)	1.657	0.799	0.830
C18	0.73 (0.58, 0.89)	0.71 (0.57, 0.90)	0.73 (0.58, 0.885)	0.75 (0.62, 0.92)	0.70 (0.57, 0.87)	5.467	0.236	0.278
C18:1	1.31 (1.08, 1.54)	1.29 (1.075, 1.54)	1.335 (1.085, 1.60)	1.35 (1.05, 1.57)	1.28 (1.07, 1.49)	5.942	0.171	0.222
C18:1OH	0.01 (0.01, 0.02)	0.01 (0.01, 0.02)	0.01 (0.01, 0.02)	0.01 (0.01, 0.02)	0.01 (0.01, 0.02)	5.460	0.200	0.254
C18:2	0.24 (0.185, 0.31)	0.24 (0.19, 0.305)	0.235 (0.19, 0.31)	0.23 (0.17, 0.30)	0.24 (0.19, 0.30)	1.693	0.813	0.830
C18OH	0.01 (0.00, 0.01)	0.01 (0.00, 0.01)	0.01 (0.00, 0.01)	0.01 (0.00, 0.01)	0.01 (0.00, 0.01)	30.79	<0.001	<0.001

FDR, false discovery fate; C0, free carnitine; C2, acetylcarnitine; C3, propionylcarnitine; C3DC + C4OH, malonylcarnitine + 3-hydroxybutyrylcarnitine; C4, butyrylcarnitine + isobutyrylcarnitine; C4DC + C5OH, methylmalonyl + 3-hydroxy-isovalerylcarnitine; C5, sovalerylcarnitine + methylbutyrylcarnitine; C5:1:tiglylcarnitine; C5DC + C6OH, glutarylcarnitine + 3- hydroxyhexanoylcarnitine; C6, hexanoylcarnitine; C6DC, methylglutarylcarnitine; C8, octanoylcarnitine; C8:1, octenoylcarnitine; C10, decanoylcarnitine; C10:1, decenoylcarnitine; C10:2, decadienoylcarnitine; C12, dodecanoylcarnitine; C12:1:dodecenoylcarnitine; C14, ltetradecanoylcarnitine; C14:1, tetradecenoylcarnitine; C14:2, tetradecadienoylcarnitine; C14OH, 3-hydroxy-tetradecanoylcarnitine; C16, palmitoylcarnitine; C16:1, palmitoleylcarnitine; C16OH, 3-hydroxy-hexadecanoylcarnitine; C16:1OH, 3-hydroxy-hexadecenoylcarnitine; C18, stearoylcarnitine; C18:1, oleoylcarnitine; C18:1OH, 3-hydroxy-octadecenoylcarnitine; C18:2, linoleoylcarnitine; C18OH, 3-hydroxy-octadecanoylcarnitine.

Pairwise comparisons of the 21 acylcarnitines across HDP subgroups and the normal pregnancy group are illustrated in Supplementary Figure S1. Newborns from the GH, PE, CH, or CH + PE subgroups exhibited higher levels of C2, C3DC + C4OH, C5DC + C6OH, C8, C10:2, C12, and C14:1 than those from the normal pregnancy group (*p* < 0.05). In addition, C3, C6, C10, C14:2, C14OH, and C16:1 levels were significantly higher in newborns from the GH, PE, or CH subgroups than in those from the normal pregnancy group. No significant differences were observed in the levels of C2, C3, C3DC + C4OH, C5:1, C5DC + C6OH, C6, C8:1, C10, C10:1, C12, C14:2, C14OH, C16, and C16:1 among the pairwise comparisons of the four HDP subgroups.

In addition, Supplementary Table S3 presents the acylcarnitine level-related differences between the HDP and normal pregnancy groups for male or female infants. We also performed the acylcarnitine and birth weight correlation analyses. The results showed that birth weight was significantly correlated with C0, C2, C5, C3DC + C4OH, C8, C10, C10:1, C10:2, C14:1, C14:2, C14OH, C16:1, C18:1, C18:2, and C18:1OH in the HDP group (*p* < 0.05) (Figure 5A), whereas only C3 was significantly correlated with birth weight in the normal pregnancy group (Figure 5B). The scatter plot of the statistically significant correlations between certain acylcarnitines and birth weight in the HDP group are presented in Figure 5C. After adjusting for gestational age and birth weight, as well as neonatal sex, the levels of C2, C8, C8:1, C10:2, and C12 were significantly higher in newborns from the GH, PE, CH, and CH + PE subgroups than in those from the normal pregnancy group (Table 6). Furthermore, C3, C3DC + C4OH, C5DC + C6OH, C6, C14:2, C14OH, and C16:1 levels were significantly higher in newborns from the GH, PE, and CH subgroups than in those from the normal pregnancy group. Adjusting for gestational age, birth weight, and neonatal sex, the overall levels of C0, C2, C8, C10:1, C18:1, C18:1OH, and C18:2 in newborns in the HDP group with FGR were higher than those in newborns in the HDP group without FGR (Supplementary Table S4).

**Table 6:** Associations between hypertensive disorders in pregnancy and the level of acylcarnitines in peripheral blood of newborns.

Acylcarnitine	Pre-eclampsia group (*N* = 363)		Gestational hypertension group (*N* = 632)		Chronic hypertension superimposed pre-eclampsia group (*N* = 57)		Chronic hypertension group (*N* = 176)	
	*β* (95% CI)	FDR*	*β* (95% CI)	FDR*	*β* (95%CI)	FDR*	*β* (95% CI)	FDR*
C0	−0.428 (−1.321 to 0.465)	0.464	−0.615 (−1.332to 0.101)	0.324	0.180 (−1.694 to 2.054)	0.851	−0.808 (−1.939 to 0.324)	0.324
C2	1.713 (0.955 to 2.471)	0.000	1.793 (1.184 to 2.401)	0.000	2.792 (1.201 to 4.383)	0.001	1.271 (0.310 to 2.232)	0.010
C3	0.088 (0.010 to 0.166)	0.036	0.107 (0.045 to 0.170)	0.003	0.041 (−0.123 to 0.204)	0.624	0.130 (0.031 to 0.228)	0.020
C3DC + C4OH	0.008 (0.002 to 0.014)	0.012	0.011 (0.006 to 0.016)	0.000	0.008 (−0.005 to 0.022)	0.205	0.012 (0.004 to 0.020)	0.007
C4	−0.001 (−0.008 to 0.007)	0.825	−0.001 (−0.007 to 0.007)	0.825	−0.006 (−0.022 to 0.010)	0.825	−0.005 (−0.015 to 0.004)	0.825
C4DC + C5OH	−0.003 (−0.009 to 0.004)	0.717	0.002 (−0.004 to 0.007)	0.717	0.002 (−0.012 to 0.016)	0.812	−0.003 (−0.012to 0.005)	0.717
C5	0.000 (−0.005 to 0.005)	0.979	−0.005 (−0.009 to −0.001)	0.037	−0.004 (−0.014 to 0.007)	0.931	0.000 (−0.006 to 0.007)	0.979
C5:1	0.000 (−0.001 to 0.001)	0.947	0.000 (0.000 to 0.001)	0.347	−0.001 (−0.002 to 0.001)	0.814	0.000 (−0.001 to 0.001)	0.947
C5DC + C6OH	0.004 (0.001 to 0.007)	0.018	0.007 (0.004 to 0.010)	0.000	0.006 (−0.002 to 0.013)	0.131	0.006 (0.002 to 0.010)	0.016
C6	0.003 (0.002 to 0.004)	0.000	0.004 (0.003 to 0.005)	0.000	0.003 (−0.001 to 0.006)	0.140	0.003 (0.001 to 0.005)	0.013
C6DC	−0.011 (−0.018 to −0.005)	0.002	−0.003 (−0.008 to 0.002)	0.357	−0.018 (−0.032 to −0.005)	0.016	−0.002 (−0.010 to 0.006)	0.600
C8	0.007 (0.005 to 0.009)	0.000	0.008 (0.006 to 0.009)	0.000	0.004 (0.000 to 0.009)	0.048	0.004 (0.002 to 0.007)	0.002
C8:1	0.019 (0.014 to 0.023)	0.000	0.015 (0.011 to 0.019)	0.000	0.011 (0.001 to 0.021)	0.035	0.007 (0.001 to 0.013)	0.035
C10	0.008 (0.005 to 0.011)	0.000	0.009 (0.007 to 0.012)	0.000	0.004 (−0.002 to 0.010)	0.188	0.005 (0.002 to 0.009)	0.008
C10:1	0.005 (0.002 to 0.007)	0.002	0.003 (0.001 to 0.005)	0.015	0.002 (−0.003 to 0.008)	0.462	0.002 (−0.001 to 0.006)	0.259
C10:2	0.001 (0.001 to 0.002)	0.000	0.002 (0.001 to 0.002)	0.000	0.001 (0.000 to 0.002)	0.010	0.001 (0.000 to 0.001)	0.022
C12	0.009 (0.006 to 0.012)	0.000	0.009 (0.007 to 0.012)	0.000	0.007 (0.001 to 0.014)	0.022	0.007 (0.004 to 0.011)	0.000
C12:1	0.001 (−0.002 to 0.005)	0.545	−0.001 (−0.003 to 0.002)	0.545	0.003 (−0.004 to 0.010)	0.545	0.002 (−0.002 to 0.006)	0.545
C14	0.001 (−0.006 to 0.008)	0.823	0.002 (−0.003 to 0.007)	0.823	0.002 (−0.013 to 0.017)	0.823	−0.001 (−0.010 to0.008)	0.823
C14:1	0.005 (0.001 to 0.008)	0.026	0.003 (0.000 to 0.006)	0.040	0.006 (0.000 to 0.013)	0.067	0.005 (0.001 to 0.009)	0.030
C14:2	0.001 (0.001 to 0.002)	0.002	0.001 (0.001 to 0.002)	0.001	0.000 (−0.001 to 0.002)	0.683	0.002 (0.001 to 0.002)	0.003
C14OH	0.001 (0.001 to 0.002)	0.000	0.001 (0.001 to 0.002)	0.000	0.001 (0.001 to 0.002)	0.115	0.001 (0.001 to 0.002)	0.013
C16	0.075 (−0.042 to 0.192)	0.282	0.191 (0.097 to 0.285)	0.000	0.114 (−0.132 to 0.360)	0.363	0.096 (−0.052 to 0.245)	0.282
C16:1	0.009 (0.003 to 0.016)	0.014	0.015 (0.009 to 0.020)	0.000	0.012 (−0.001 to 0.026)	0.080	0.011 (0.002 to 0.019)	0.018
C16OH	0.000 (0.000 to o.001)	0.684	0.001 (0.000 to 0.001)	0.015	0.001 (−0.001 to 0.002)	0.452	0.000 (0.000 to 0.001)	0.385
C16:1OH	0.000 (−0.001 to 0.002)	0.949	0.000 (−0.002 to 0.001)	0.949	−0.001 (−0.005 to 0.003)	0.949	0.000 (−0.002 to 0.002)	0.949
C18	0.014 (−0.018 to 0.046)	0.526	0.026 (0.000 to 0.051)	0.200	0.022 (−0.045 to 0.088)	0.526	0.016 (−0.024 to 0.057)	0.526
C18:1	0.036 (−0.012 to 0.084)	0.187	0.045 (0.006 to 0.083)	0.090	0.031 (−0.070 to 0.132)	0.546	0.055 (−0.006 to 0.116)	0.151
C18:1OH	0.000 (0.000 to 0.001)	0.374	0.000 (0.000 to 0.001)	0.389	0.001 (−0.001 to 0.002)	0.399	0.001 (0.000 to 0.002)	0.325
C18:2	0.005 (−0.006 to 0.017)	0.479	0.004 (−0.005 to 0.013)	0.479	−0.009 (−0.033 to 0.015)	0.479	0.005 (−0.009 to 0.020)	0.479
C18OH	0.001 (0.000 to 0.001)	0.031	0.001 (0.001 to 0.002)	0.000	0.001 (0.000 to 0.003)	0.107	0.000 (0.000 to 0.001)	0.312

FDR, false discovery fate; C0, free carnitine; C2, acetylcarnitine; C3, propionylcarnitine; C3DC + C4OH, malonylcarnitine + 3-hydroxybutyrylcarnitine; C4, butyrylcarnitine + isobutyrylcarnitine; C4DC + C5OH, methylmalonyl + 3-hydroxy-isovalerylcarnitine; C5, sovalerylcarnitine + methylbutyrylcarnitine; C5:1:tiglylcarnitine; C5DC + C6OH, glutarylcarnitine + 3- hydroxyhexanoylcarnitine; C6, hexanoylcarnitine; C6DC, methylglutarylcarnitine; C8, octanoylcarnitine; C8:1, octenoylcarnitine; C10, decanoylcarnitine; C10:1, decenoylcarnitine; C10:2, decadienoylcarnitine; C12, dodecanoylcarnitine; C12:1:dodecenoylcarnitine; C14, ltetradecanoylcarnitine; C14:1, tetradecenoylcarnitine; C14:2, tetradecadienoylcarnitine; C14OH, 3-hydroxy-tetradecanoylcarnitine; C16, palmitoylcarnitine; C16:1, palmitoleylcarnitine; C16OH, 3-hydroxy-hexadecanoylcarnitine; C16:1OH, 3-hydroxy-hexadecenoylcarnitine; C18, stearoylcarnitine; C18:1, oleoylcarnitine; C18:1OH, 3-hydroxy-octadecenoylcarnitine; C18:2, linoleoylcarnitine; C18OH, 3-hydroxy-octadecanoylcarnitine.

The models were adjusted for gestational age at delivery, birth weight, and neonatal sex. *FDR was calculated to account for multi- ple comparisons using the Benjamini-Hochberg method.

**Figure 6:**
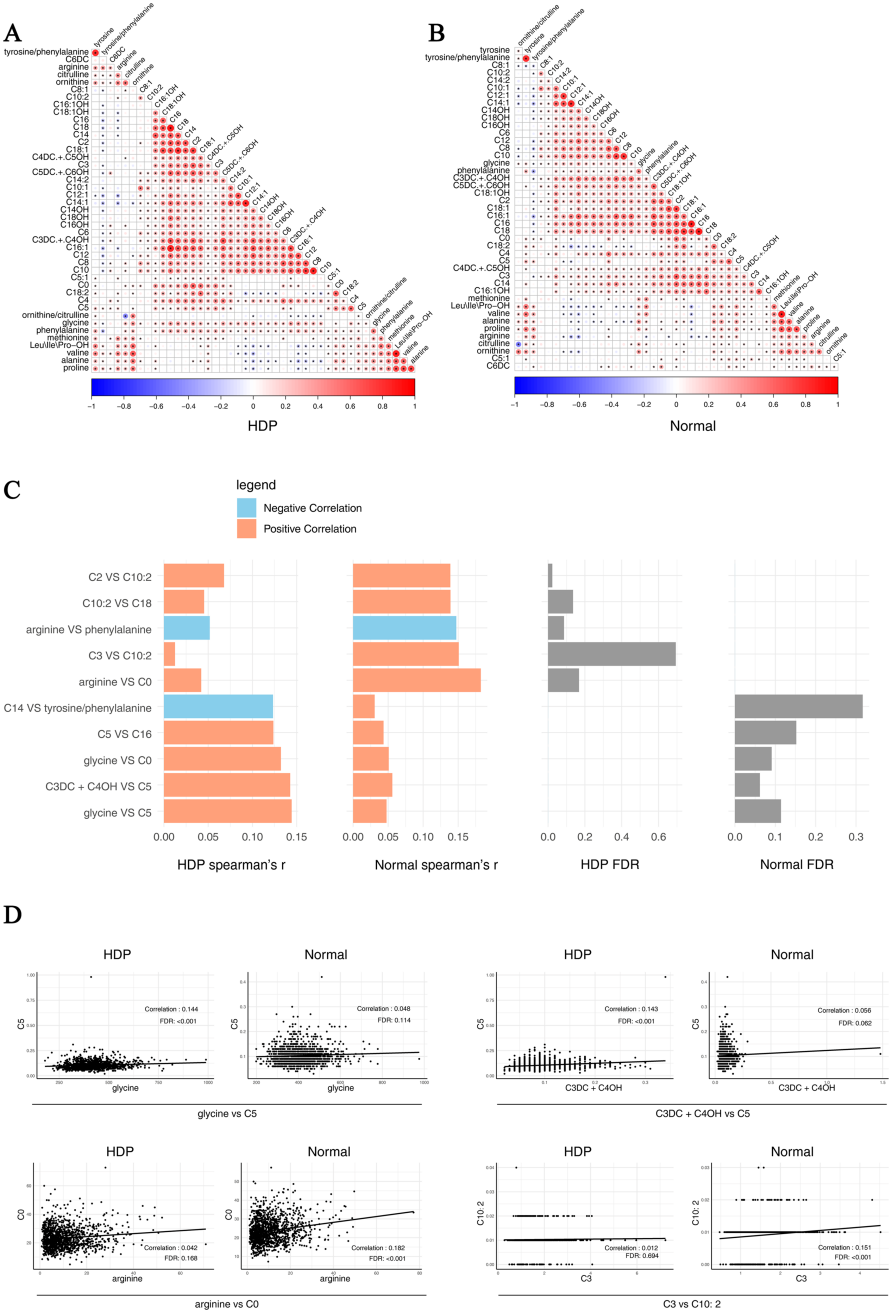
Pairwise correlations across metabolites in the HDP and normal pregnancy groups. Correlations specific to each group are highlighted by a rectangle. **(A)** Pairwise correlations across metabolites in the HDP group. **(B)** Pairwise correlations across metabolites in the normal pregnancy group. **(C)** The top 10 different pairwise correlations across metabolites between the HDP and normal pregnancy groups. **(D)** The scatter plot of different pairwise correlations between the HDP and normal pregnancy groups. HDP, hypertensive disorders in pregnancy; Leu\Ile\Pro-OH, leucine\isoleucine\hydroxyproline; C0, free carnitine; C2, acetylcarnitine; C3, propionylcarnitine; C3DC + C4OH, malonylcarnitine + 3-hydroxybutyrylcarnitine; C4, butyrylcarnitine + isobutyrylcarnitine; C4DC + C5OH, methylmalonyl + 3-hydroxy-isovalerylcarnitine; C5, sovalerylcarnitine + methylbutyrylcarnitine; C5:1, tiglylcarnitine; C5DC + C6OH, glutarylcarnitine + 3- hydroxyhexanoylcarnitine; C6, hexanoylcarnitine; C6DC, methylglutarylcarnitine; C8, octanoylcarnitine; C8:1, octenoylcarnitine; C10, decanoylcarnitine; C10:1, decenoylcarnitine; C10:2, decadienoylcarnitine; C12, dodecanoylcarnitine; C12:1, dodecenoylcarnitine; C14, ltetradecanoylcarnitine; C14:1, tetradecenoylcarnitine; C14:2, tetradecadienoylcarnitine; C14OH, 3-hydroxy-tetradecanoylcarnitine; C16, palmitoylcarnitine; C16:1, palmitoleylcarnitine; C16OH, 3-hydroxy-hexadecanoylcarnitine; C16:1OH, 3-hydroxy-hexadecenoylcarnitine; C18, stearoylcarnitine; C18:1, oleoylcarnitine; C18:1OH, 3-hydroxy-octadecenoylcarnitine; C18:2, linoleoylcarnitine; C18OH, 3-hydroxy-octadecanoylcarnitine.

In addition, we analyzed pairwise correlations across metabolites in the HDP and normal pregnancy groups. The results are presented in Figures 6A,B. The top 10 different pairwise correlations across metabolites between the HDP and normal pregnancy groups are shown in Figure 6C. Meanwhile, the scatter plot of certain different pairwise correlations between the HDP and normal pregnancy groups are presented in Figure 6D.

## 4 Discussion

Expanded NBS, which incorporates MS/MS technology, offers a fundamental metabolic profile for newborns and serves as a panel of biomarkers indicative of fetal growth and overall health. Numerous fetal and maternal factors may influence the metabolites detected through NBS. However, beyond gestational age and birth weight, the understanding of the underlying etiologies affecting these measurements at birth remains limited. This study utilized a targeted metabolomics platform to analyze the blood metabolites of newborns born to mothers with HDP using MS/MS analysis. Our findings revealed a significant difference in blood metabolism between newborns of mothers with HDP and those of mothers with normal pregnancies, suggesting distinct metabolic characteristics.

During pregnancy, glucose and amino acids, particularly arginine, play vital roles in energy metabolism and fetal growth. They significantly regulate the mechanistic target of rapamycin (mTOR), which is associated with cell growth and proliferation, ultimately contributing to enhanced fetal development (46). Beyond serving as a building block for tissue proteins, arginine functions as a physiological precursor for synthesizing essential molecules such as creatine and polyamines (47). Arginine is also a precursor for nitric oxide (NO), which is critical for vasodilation and adequate placental blood flow. Lower arginine levels lead to placental insufficiency and precipitate endothelial dysfunction. Notably, dysfunction in the arginine-NO pathway may impair interneuronal communication (48, 49). Numerous studies have established a close association between PE and neurodevelopmental disorders in the offspring (13, 14, 50, 51). Additionally, arginine deficiency can lead to hyperammonemia and cardiovascular, pulmonary, intestinal, immunological, and neurological dysfunctions, particularly in preterm infants (52). Our study revealed significantly lower arginine concentrations in newborns of mothers with PE than in those of mothers with normal pregnancies. However, alterations in arginine levels in newborns born to mothers with PE-induced neurodevelopmental disorders require further exploration.

The primary metabolic pathway for phenylalanine, an essential amino acid, is its conversion to tyrosine by phenylalanine hydroxylase. Placental insufficiency causes accumulation of phenylalanine because it is less readily metabolized to tyrosine. Increased levels of phenylalanine may indicate a malfunction in the transport of amino acids and are frequently observed in fetal growth restriction (53). In this study, tyrosine concentrations were significantly lower in infants born to mothers with PE, GH, and CH + PE than in those born to mothers with normal pregnancies, consistent with previous findings (54, 55). Tyrosine is crucial for synthesizing monoamine neurotransmitters, which play a crucial role in signaling within the central and peripheral nervous systems and are involved in the regulation of various neurocognitive and motor functions (56).

Branched-chain amino acids (BCAAs) include three common protein amino acids: leucine, valine, and isoleucine. The main function of BCAAs is to promote anabolic metabolism, such as muscle growth, by stimulating the release of insulin and growth hormones. Leucine acts as a signaling molecule, which enhances protein synthesis in peripheral tissues by activating the mTOR complex 1 pathway. This pathway is the primary regulator of cell growth and proliferation, particularly in the skeletal muscle, which constitutes 30% of the neonatal body mass and is the fastest-growing body compartment (57, 58). Severe PE can lead to several adverse fetal outcomes, such as intrauterine growth restriction (59, 60). Placental transport of BCAAs is impaired in hypertensive states due to placental insufficiency and reduced activity of amino acid transporters (System L and System A) (61). Intrauterine growth restriction and compromised fetal development are linked to low BCAA levels (62). In this study, the overall levels of Leu\Ile\Pro-OH and valine in newborns in the HDP group were lower than those in newborns in the normal pregnancy group. This difference may partially explain the increased risk of fetal growth restriction in women with HDP.

Most ammonia in the body is converted to urea through the ornithine cycle to eliminate the toxic effects of ammonia. In the mitochondria, carbamoyl phosphate and ornithine are catalyzed by ornithine transcarbamylase to produce citrulline, marking the initiation of the urea cycle. Ornithine serves as a byproduct of L-alanine synthesis via arginase, which is an important step in the urea cycle. Studies have indicated that changes in amino acid levels, particularly in the urea cycle, may play a significant role in PE development (63, 64). In line with these findings, our study revealed significant differences in the levels of ornithine and citrulline, as well as the ratio of ornithine/citrulline, in newborns from the HDP group compared to those in newborns from the normal pregnancy group.

Methionine is a sulfur-containing amino acid necessary for protein synthesis and the production of vital nutrients via the methionine cycle. This cycle transfers the terminal methyl group from methionine to various methylated products, resulting in homocysteine formation. Subsequently, homocysteine irreversibly donates sulfur atoms for cysteine synthesis (65). Deficits in methionine may alter epigenetic regulation and fetal growth (66). Studies have shown that elevated homocysteine and methionine levels in pregnant women with PE can lead to oxidative stress, an important pathological mechanism underlying the occurrence of PE (67, 68). In this study, the levels of MET in newborns in the GH, PE, and CH groups were significantly higher than those in newborns in the normal pregnancy group, consistent with the findings of a previous study (54).

Alanine is a glucogenic amino acid that facilitates glucose metabolism, alleviates hypoglycemia, and enhances energy levels in the body. Alanine aminotransferase catalyzes the reversible transamination between pyruvate and glutamate to produce alanine and 2-oxoglutarate, thereby contributing to gluconeogenesis (69). Alanine deficiency is associated with hypoglycemia, whereas elevated alanine levels are associated with an increased risk of gout and diabetes (70). A previous study revealed that hypoglycemia rates were significantly higher in infants born to mothers with PE than in those born to mothers with normal pregnancies (71). Placental insufficiency reduces alanine transport to the fetus. Reduced alanine levels may imply inadequate fetal energy generation (72). In the present study, blood ALA concentrations were significantly lower in the HDP group than in the normal pregnancy group.

Non-essential amino acids—serine and glycine—are elevated as a defense mechanism against oxidative stress and compromised placental transport. Elevated serine indicates altered amino acid metabolism, while higher glycine levels are linked to fetal growth restriction (73). Glycine is synthesized from choline, serine, Pro-OH, and threonine through inter-organ metabolic processes (74). It serves as a precursor for several important metabolites, including glutathione, purines, heme, and creatine, and plays a crucial role in gluconeogenesis. Additionally, glycine plays an important role in antioxidant, anti-inflammatory, and immunomodulatory effects in both peripheral and nervous tissues (75).

Proline is a non-essential amino acid, and its concentration in the blood has been positively correlated with the birth weight of newborns (76). Decreased proline levels have been identified as a risk factor for low birth weight in newborns born to mothers with HDP compared with that in newborns born to mothers with normal pregnancies (38). Our study also revealed that proline concentrations were significantly lower in infants born to mothers with HDP than in those born to mothers with normal pregnancies.

The observed changes in acylcarnitine levels are likely attributed to a complex interplay of maternal, fetal, and placental factors. Studies have suggested that women with PE may exhibit elevated plasma acylcarnitine levels, potentially resulting from abnormalities in fatty acid oxidation, renal function, dyslipidemia, hemoconcentration, and oxidative stress (55, 77–79). Elevated acylcarnitine levels in maternal circulation can be transferred to fetal circulation through the placenta, which can be detected through NBS shortly after birth. Furthermore, a previous study revealed that the levels of carnitine and short-, medium-, and long-chain acylcarnitines in cord plasma were significantly higher in the PE group than in the healthy pregnancy group (80). In addition, elevated levels of carnitine precursors and trimethylated compounds in the cord plasma have been associated with PE at birth (81). Therefore, the transfer of amino acids and acylcarnitines from maternal-fetal blood to cord blood is higher than that in maternal blood (80). Alternatively, PE may act as a stressor for neonates, activating the fatty acid oxidation pathway, which serves as a nonspecific marker of the catabolic state. A previous study showed that the levels of C0, C2, C8:1, and C18:2 were significantly elevated in infants born to mothers with PE compared to those in infants born to mothers without PE (55). Thus, neonatal acylcarnitine profiles in hypertensive pregnancies reveal metabolic stress, disturbed fatty acid oxidation, and compromised mitochondrial activity. Placental insufficiency, hypoxia, and oxidative stress are the main causes of these alterations, leading to less energy production by the fetus. In our study, the levels of short-, medium-, and long-chain acylcarnitines were significantly higher in newborns in the HDP group than in those in the normal pregnancy group.

This study draws attention to the metabolic effects of HDP in neonates and provides a framework for further investigation of the mechanisms, clinical results, and possible treatments. It provides data support and strategies for future fetal intrauterine screening and management; early identification and screening of at-risk neonates; and postnatal potential interventions, monitoring strategies, and follow up. Furthermore, the targeted amino acids and acylcarnitines in the dried blood using MS/MS, characterized by high sensitivity, specificity, and simplicity, demonstrate its applicability in routine practice.

While the size of the study cohort is big (2,456 subjects), some limitations should be acknowledged. First, as a retrospective case–control study, it may be susceptible to selection bias. Second, this study was conducted in a single geographic region, and thus results might not be generalizable to other populations. Third, maternal-fetal correlations for amino acid and acylcarnitine concentrations could not be established because samples were obtained solely from infants. Further, we did not collect data on placental pathology and maternal blood or urine biomarkers of inflammation, oxidative stress, or endothelial dysfunction. Therefore, future research should focus on elucidating several key areas to enhance the understanding of the maternal-fetal correlations regarding amino acid and acylcarnitine concentrations, as well as the influence of maternal BP trajectories throughout pregnancy on neonatal amino acid and acylcarnitine levels. In addition, fetal intrauterine screening and management through targeted metabolomics, as well as early identification and screening of high-risk newborns and subsequent follow-up, should be explored in future research.

In conclusion, this study revealed a significant association between HDP and neonatal amino acid and acylcarnitine levels, which were mainly involved in alanine and proline metabolism, the urea cycle pathway, and the fatty acid oxidation pathway (Figure 1).

## Data Availability

The original contributions presented in the study are included in the article/Supplementary material, further inquiries can be directed to the corresponding authors.

## Footnotes

^1^ https://www.r-project.org/

## Supplementary material

SUPPLEMENTARY FIGURE S1 Pairwise comparisons of the levels of acylcarnitines among the subgroups within the hypertensive disorders in pregnancy and normal pregnancy groups. Owing to the low detection values of most acylcarnitines, this leads to overlapping data points. **(A)** C2; **(B)** C3; **(C)** C3DC + C4OH; **(D)** C4DC + C5OH; **(E)** C5:1; **(F)** C5DC + C6OH; **(G)** C6; **(H)** C6DC; **(I)** C8; **(J)** C8:1; **(K)** C10; **(L)** C10:1; **(M)** C10:2; **(N)** C12; **(O)** C14:1; **(P)** C14:2; **(Q)** C14OH; **(R)** C16OH; **(S)** C16:1; **(T)** C16; **(U)** C18OH. Data are presented as median with interquartile range. **p* < 0.05, ***p* < 0.01, ****p* < 0.001, *****p* < 0.0001. C2, acetylcarnitine; C3, propionylcarnitine; C3DC + C4OH, malonylcarnitine + 3-hydroxybutyrylcarnitine; C4DC + C5OH, methylmalonyl + 3-hydroxy-isovalerylcarnitine; C5:1, tiglylcarnitine; C5DC + C6OH, glutarylcarnitine + 3- hydroxyhexanoylcarnitine; C6, hexanoylcarnitine; C6DC, methylglutarylcarnitine; C8, octanoylcarnitine; C8:1, octenoylcarnitine; C10, decanoylcarnitine; C10:1, decenoylcarnitine; C10:2, decadienoylcarnitine; C12, dodecanoylcarnitine; C14:1, tetradecenoylcarnitine; C14:2, tetradecadienoylcarnitine; C14OH, 3-hydroxy-tetradecanoylcarnitine; C16OH, 3-hydroxy-hexadecanoylcarnitine; C16:1, palmitoleylcarnitine; C16, palmitoylcarnitine; C18OH, 3-hydroxy-octadecanoylcarnitine; PE, pre-eclampsia; CH, chronic hypertension; CH + PE, chronic hypertension superimposed pre-eclampsia.

## Glossary

BCAAs: Branched-chain amino acids
BP: blood pressure
C2: acetylcarnitine
C3: propionylcarnitine
C3DC + C4OH: malonylcarnitine + 3-hydroxybutyrylcarnitine
C4DC + C5OH: methylmalonyl + 3-hydroxy-isovalerylcarnitine
C5:1: tiglylcarnitine
C5DC + C6OH: glutarylcarnitine + 3- hydroxyhexanoylcarnitine
C6: hexanoylcarnitine
C6DC: methylglutarylcarnitine
C8: octanoylcarnitine
C8:1: oc-tenoylcarnitine
C10: decanoylcarnitine
C10:1: decenoylcarnitine
C10:2: decadienoylcarnitine
C12: dodeca-noylcarnitine
C14:1: tetradecenoylcarnitine
C14:2: tetradecadienoylcarnitine
C14OH: 3-hydroxy-tetradecanoylcarnitine
C16OH: 3-hydroxy-hexadecanoylcarnitine
C16:1: palmitoleylcarnitine
C16: palmitoylcarnitine
C18OH: 3-hydroxy-octadecanoylcarnitin
CH: chronic hypertension
CH + PE: chronic hypertension with superimposed pre-eclampsia
DBP: diastolic blood pressure
GH: gestational hypertension
HDP: hypertensive disorders in pregnancy
ILE: isoleucine
Leu: leucine
MS: mass spectrometry
mTOR: mechanistic target of rapamycin
NBS: newborn screening
NO: nitric oxide
PE: pre-eclampsia
Pro-OH: hydroxyproline
SBP: systolic blood pressure
