# Supplementary figures and images for: The influence of hypertensive disorders in pregnancy on neonatal amino acid and acylcarnitine levels
Source: Front Nutr. 2025 Aug 15;12:1520262. doi: 10.3389/fnut.2025.1520262 (PMC12402939; doi:10.3389/fnut.2025.1520262)

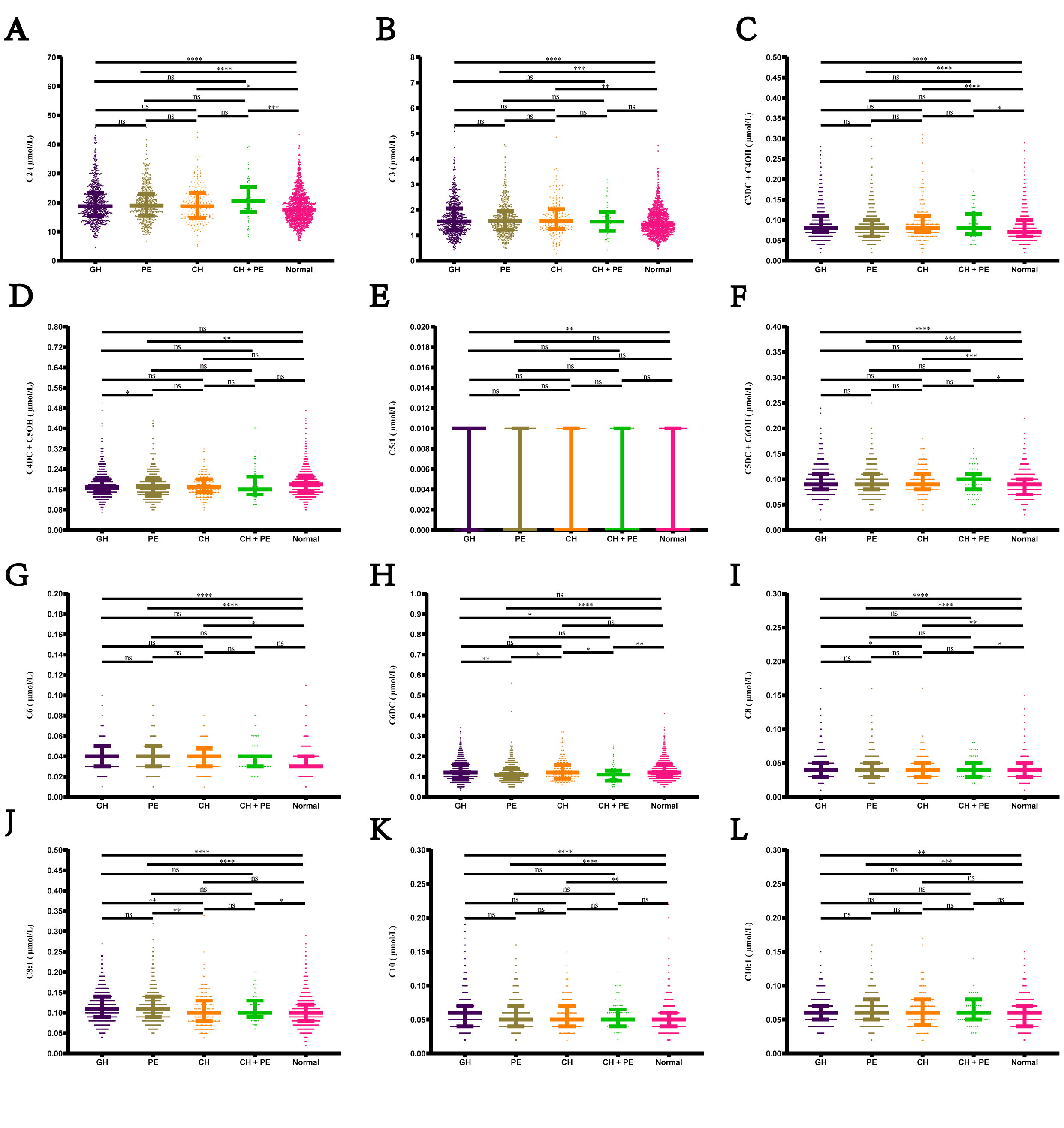

Supplement: SUPPLEMENTARY FIGURE S1 — Pairwise comparisons of the levels of acylcarnitines among the subgroups within the hypertensive disorders in pregnancy and normal pregnancy groups. Owing to the low detection values of most acylcarnitines, this leads to overlapping data points. (A) C2; (B) C3; (C) C3DC + C4OH; (D) C4DC + C5OH; (E) C5:1; (F) C5DC + C6OH; (G) C6; (H) C6DC; (I) C8; (J) C8:1; (K) C10; (L) C10:1; (M) C10:2; (N) C12; (O) C14:1; (P) C14:2; (Q) C14OH; (R) C16OH; (S) C16:1; (T) C16; (U) C18OH. Data are presented as median with interquartile range. *p < 0.05, **p < 0.01, ***p < 0.001, ****p < 0.0001. C2, acetylcarnitine; C3, propionylcarnitine; C3DC + C4OH, malonylcarnitine + 3-hydroxybutyrylcarnitine; C4DC + C5OH, methylmalonyl + 3-hydroxy-isovalerylcarnitine; C5:1, tiglylcarnitine; C5DC + C6OH, glutarylcarnitine + 3- hydroxyhexanoylcarnitine; C6, hexanoylcarnitine; C6DC, methylglutarylcarnitine; C8, octanoylcarnitine; C8:1, octenoylcarnitine; C10, decanoylcarnitine; C10:1, decenoylcarnitine; C10:2, decadienoylcarnitine; C12, dodecanoylcarnitine; C14:1, tetradecenoylcarnitine; C14:2, tetradecadienoylcarnitine; C14OH, 3-hydroxy-tetradecanoylcarnitine; C16OH, 3-hydroxy-hexadecanoylcarnitine; C16:1, palmitoleylcarnitine; C16, palmitoylcarnitine; C18OH, 3-hydroxy-octadecanoylcarnitine; PE, pre-eclampsia; CH, chronic hypertension; CH + PE, chronic hypertension superimposed pre-eclampsia. [file Image_1.tif]

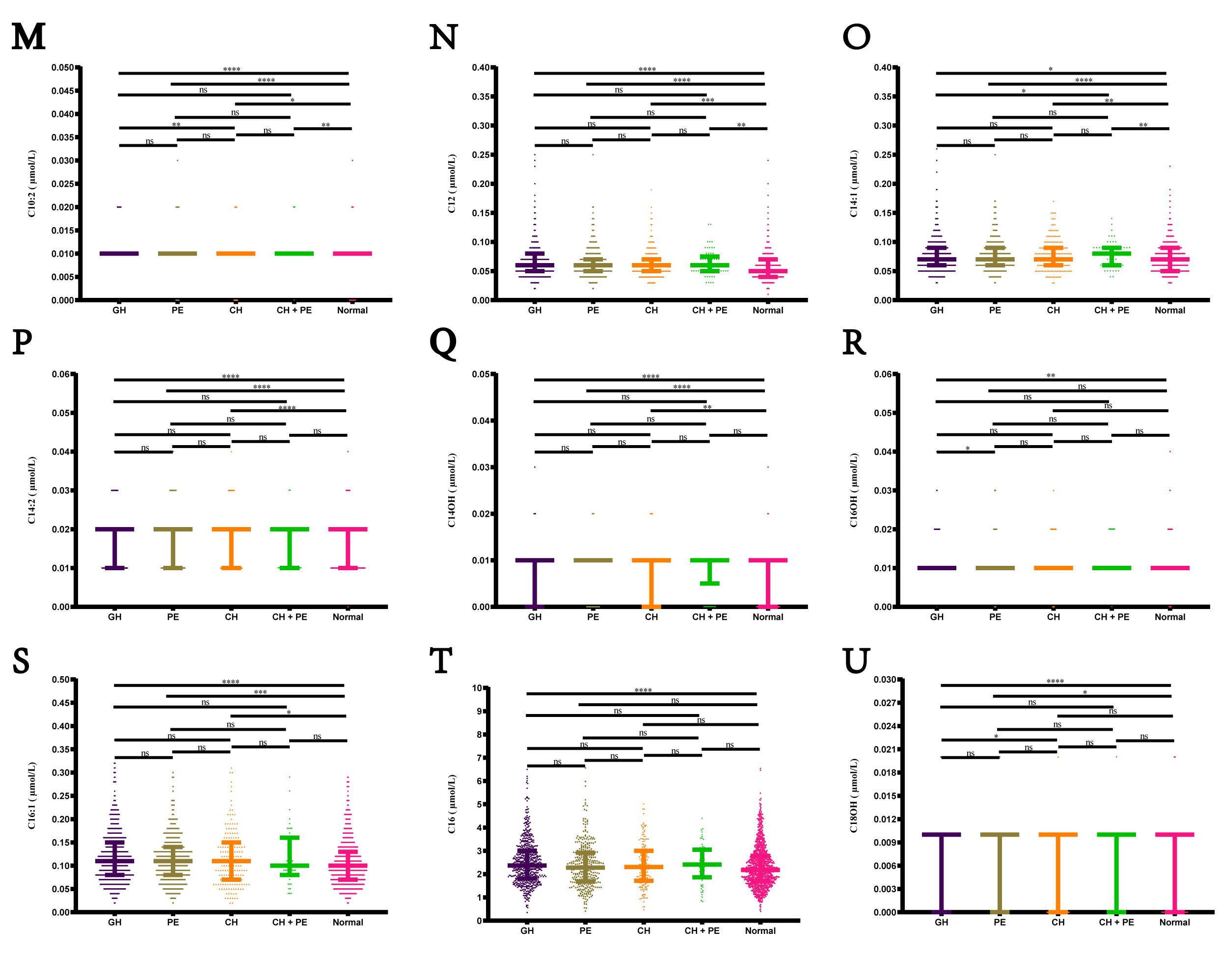

Supplement: Supplementary file 2 [file Image_2.tif]
