# Supplementary material for: The influence of hypertensive disorders in pregnancy on neonatal amino acid and acylcarnitine levels
Source: Front Nutr. 2025 Aug 15;12:1520262. doi: 10.3389/fnut.2025.1520262 (PMC12402939; doi:10.3389/fnut.2025.1520262)
Supplement: Supplementary file 3 [file Table_1.docx]

**Table S1. The parameters of the liquid tandem mass spectrometer**

**Table S2. The results of PERMANOVA analysis between the hypertensive disorders in pregnancy group and the normal pregnancy group**

**Table S3. The sex-stratified analyses of amino acid and acylcarnitine levels in peripheral blood of newborns between the hypertensive disorders in pregnancy group and the normal pregnancy group**

**Table S4. The FGR-stratified analyses of amino acid and acylcarnitine levels in peripheral blood of newborns in the hypertensive disorders in pregnancy group**

**Table S1. The parameters of the liquid tandem mass spectrometer**

| Compound Nam | Parent (m/z) | Daughter (m/z) | Dwell (s) | Cone (v) | Collision (v) |
| --- | --- | --- | --- | --- | --- |
| glycine | 76 | 30 | 0.050 | 20 | 7 |
| glycine IS | 78 | 32 | 0.050 | 20 | 7 |
| alanine | 90.1 | 44 | 0.050 | 20 | 8 |
| alanine IS | 94.1 | 48 | 0.050 | 20 | 8 |
| proline | 116.1 | 70.1 | 0.025 | 12 | 14 |
| proline IS | 121.1 | 74.1 | 0.025 | 12 | 14 |
| valine | 118.1 | 72.1 | 0.025 | 22 | 12 |
| valine IS | 126.1 | 80.1 | 0.025 | 22 | 12 |
| Leu/Ile/Pro-OH | 132.1 | 86.1 | 0.025 | 21 | 10 |
| Leu/Ile/Pro-OH IS | 135.1 | 89.1 | 0.025 | 21 | 10 |
| ornithine | 133.2 | 70.1 | 0.025 | 18 | 16 |
| ornithine IS | 139.2 | 76.1 | 0.025 | 18 | 16 |
| methionine | 150.1 | 104.1 | 0.025 | 19 | 10 |
| methionine IS | 153.1 | 107.1 | 0.025 | 19 | 10 |
| phenylalanine | 166.1 | 120.1 | 0.025 | 22 | 13 |
| phenylalanine IS | 172.1 | 126.1 | 0.025 | 22 | 13 |
| citrulline | 176.1 | 113.1 | 0.025 | 19 | 16 |
| citrulline IS | 178.1 | 115.1 | 0.025 | 19 | 16 |
| arginine | 175.2 | 70.1 | 0.025 | 28 | 20 |
| arginine IS | 180.2 | 75.1 | 0.025 | 28 | 20 |
| tyrosine | 182.1 | 136.1 | 0.025 | 21 | 13 |
| tyrosine IS | 188.1 | 142.1 | 0.025 | 21 | 13 |
| C0 | 162.2 | 103 | 0.025 | 32 | 15 |
| C0 IS | 171.2 | 103 | 0.025 | 32 | 15 |
| C2 | 204.2 | 85 | 0.025 | 29 | 18 |
| C2 IS | 207.2 | 85 | 0.025 | 29 | 18 |
| C3 | 218.2 | 85 | 0.025 | 29 | 19 |
| C3 IS | 221.2 | 85 | 0.025 | 29 | 19 |
| C4 | 232.2 | 85 | 0.025 | 30 | 19 |
| C4 IS | 235.2 | 85 | 0.025 | 30 | 19 |
| C5:1 | 244.2 | 85 | 0.025 | 30 | 21 |
| C5 | 246.2 | 85 | 0.025 | 30 | 21 |
| C3DC/C4OH | 248.2 | 85 | 0.025 | 30 | 19 |
| C5 IS | 255.2 | 85 | 0.025 | 30 | 21 |
| C6 | 260.2 | 85 | 0.025 | 31 | 21 |
| C4DC/C5OH | 262.2 | 85 | 0.025 | 30 | 21 |
| C6 IS | 263.2 | 85 | 0.025 | 31 | 21 |
| C5DC/C6OH | 276.2 | 85 | 0.05 | 31 | 22 |

| C5DC/C6OH IS | 282.2 | 85 | 0.05 | 31 | 22 |
| --- | --- | --- | --- | --- | --- |
| C8:1 | 286.3 | 85 | 0.025 | 33 | 22 |
| C8 | 288.3 | 85 | 0.025 | 33 | 22 |
| C6DC | 290.2 | 85 | 0.050 | 31 | 22 |
| C8 IS | 291.3 | 85 | 0.025 | 33 | 22 |
| C10:2 | 312.3 | 85 | 0.025 | 37 | 24 |
| C10:1 | 314.3 | 85 | 0.025 | 37 | 24 |
| C10 | 316.3 | 85 | 0.025 | 37 | 24 |
| C10 IS | 319.3 | 85 | 0.025 | 37 | 24 |
| C12:1 | 342.3 | 85 | 0.025 | 40 | 26 |
| C12 | 344.3 | 85 | 0.025 | 40 | 26 |
| C12 IS | 347.3 | 85 | 0.025 | 40 | 26 |
| C14:2 | 368.3 | 85 | 0.025 | 43 | 27 |
| C14:1 | 370.3 | 85 | 0.025 | 43 | 27 |
| C14 | 372.3 | 85 | 0.025 | 43 | 27 |
| C14 IS | 375.3 | 85 | 0.025 | 43 | 27 |
| C14OH | 388.3 | 85 | 0.025 | 43 | 27 |
| C16:1 | 398.4 | 85 | 0.025 | 45 | 28 |
| C16 | 400.4 | 85 | 0.025 | 45 | 28 |
| C16 IS | 403.4 | 85 | 0.025 | 45 | 28 |
| C16:1OH | 414.4 | 85 | 0.025 | 45 | 28 |
| C16OH | 416.4 | 85 | 0.025 | 45 | 28 |
| C18:2 | 424.4 | 85 | 0.025 | 48 | 29 |
| C18:1 | 426.4 | 85 | 0.025 | 48 | 29 |
| C18 | 428.4 | 85 | 0.025 | 48 | 29 |
| C18 IS | 431.4 | 85 | 0.025 | 48 | 29 |
| \| MS/MS condition \| \| --- \| | | 3 |  |  |  |
| Capillary(KV) | | 3 |  |  |  |
| Extractor(V) | | 0.1 |  |  |  |
| RF Lens(V) | | 120 |  |  |  |
| Source Temp(℃) | | 350 |  |  |  |
| Desolvation Temp(℃) | | 650 |  |  |  |
| Desolvation Gas Flow(L/hr) | | 20 |  |  |  |
| Cone Gas Flow(L/hr) | | 3 |  |  |  |
| LC Conditions | |  |  |  |  |
| Liquid Phase Gradient | | Mobile Phase 100% (Pump A or Pump B); 0.00-0.18 min,0.20mL/min;0.18-1.00min,0.02mL/min;1.00-1.5min,0.70mL/min. | | | |
| Washes | | 50/50 methanol/Water | | | |
| Inject volume | | 20μL |  |  |  |

Abbreviation: Leu\Ile\Pro-OH: leucine\isoleucine\hydroxyproline, C0: free carnitine, C2: acetylcarnitine, C3: propionylcarnitine, C3DC + C4OH: malonylcarnitine + 3-hydroxybutyrylcarnitine, C4: butyrylcarnitine + isobutyrylcarnitine, C4DC + C5OH: methylmalonyl + 3-hydroxy-isovalerylcarnitine, C5: sovalerylcarnitine + methylbutyrylcarnitine, C5:1:tiglylcarnitine, C5DC + C6OH: glutarylcarnitine + 3- hydroxyhexanoylcarnitine, C6: hexanoylcarnitine, C6DC: methylglutarylcarnitine, C8: octanoylcarnitine, C8:1: octenoylcarnitine, C10: decanoylcarnitine, C10:1: decenoylcarnitine, C10:2: decadienoylcarnitine, C12: dodecanoylcarnitine , C12:1:dodecenoylcarnitine, C14: ltetradecanoylcarnitine, C14:1: tetradecenoylcarnitine, C14:2: tetradecadienoylcarnitine, C14OH: 3-hydroxy-tetradecanoylcarnitine, C16: palmitoylcarnitine, C16:1: palmitoleylcarnitine, C16OH: 3-hydroxy-hexadecanoylcarnitine, C16:1OH: 3-hydroxy-hexadecenoylcarnitine, C18: stearoylcarnitine, C18:1: oleoylcarnitine, C18:1OH: 3-hydroxy-octadecenoylcarnitine, C18:2 :linoleoylcarnitine, C18OH: 3-hydroxy-octadecanoylcarnitine.

**Table S2. The results of PERMANOVA analysis between the hypertensive disorders in pregnancy group and the normal pregnancy group**

|  | **Df** | **SumOfSqs** | **R2** | **F** | **Pr(>F)** |
| --- | --- | --- | --- | --- | --- |
| Group | 1 | 0.140437454 | 0.00506807 | 13.02524256 | 0.001 |
| Birth weight | 1 | 0.051547474 | 0.001860232 | 4.780906601 | 0.008 |
| Gestational age at delivery | 1 | 0.410755809 | 0.014823248 | 38.09663231 | 0.001 |
| Gender | 1 | 0.097634829 | 0.00352342 | 9.055400069 | 0.001 |
| Residual | 2451 | 26.41576619 | 0.953285221 |  |  |
| Total | 2455 | 27.71024413 | 1 |  |  |

**Table S3. The sex-stratified analyses of amino acid and acylcarnitine levels in peripheral blood of newborns between the hypertensive disorders in pregnancy group and the normal pregnancy group**

| **Amino acid or Acylcarnitine** | **Male** | | | | | **Female** | | | | |  |
| --- | --- | --- | --- | --- | --- | --- | --- | --- | --- | --- | --- |
|  | **HDP group**  **(N=626)**  **Media (P25, P75)** | **Normal group**  **(N=662)**  **Media (P25, P75)** | **Z-Value** | ***P*** | **FDR** | **HDP group**  **(N=602)**  **Media (P25, P75)** | **Normal group**  **(N=568)**  **Media (P25, P75)** | **Z-Value** | ***P*** | **FDR** |  |
| alanine | 294.19(247.03, 351.95) | 317.12(270.88, 368.95) | -5.388 | **<0.001** | **<0.001** | 304.22(252.37, 364.48) | 333.45(286.90, 391.78) | -6.687 | **<0.001** | **<0.001** |  |
| arginine | 8.41(4.90, 14.21) | 9.50(5.29, 15.95) | -2.093 | **0.036** | 0.053 | 8.08(5.05, 14.69) | 8.16(4.84, 14.11) | -0.371 | 0.711 | 0.759 |  |
| citrulline | 10.67(8.86, 13.12) | 11.73(9.70, 14.16) | -5.373 | **<0.001** | **<0.001** | 11.13(9.26, 13.53) | 11.63(9.81, 14.55) | -3.186 | **0.001** | **0.003** |  |
| glycine | 394.65(344.51, 445.22) | 390.25(340.11, 451.61) | -0.897 | 0.370 | 0.404 | 414.83(365.63, 475.11) | 404.27(360.30, 463.39) | -2.236 | **0.025** | **0.041** |  |
| Leu\Ile\  Pro-OH | 152.33(127.51, 179.62) | 163.89(139.24, 186.78) | -5.084 | **<0.001** | **<0.001** | 152.48(130.66, 175.38) | 166.37(143.55, 194.37) | -7.227 | **<0.001** | **<0.001** |  |
| methionine | 23.22(19.82, 27.47) | 22.28(19.00, 25.93) | -3.566 | **<0.001** | **<0.001** | 24.46(20.79, 28.19) | 23.49(20.18, 27.19) | -2.575 | **0.010** | **0.018** |  |
| ornithine | 102.50(83.58, 123.80) | 122.34(100.65, 147.35) | -10.244 | **<0.001** | **<0.001** | 103.80(85.64, 124.41) | 120.98(100.32, 148.10) | -9.024 | **<0.001** | **<0.001** |  |
| phenylalanine | 50.94(44.58, 58.42) | 50.18(43.79, 57.63) | -1.452 | 0.147 | 0.186 | 50.15(43.93, 57.33) | 50.73(44.28, 58.13) | -0.895 | 0.371 | 0.447 |  |
| proline | 163.23(141.30, 190.36) | 180.61(155.95, 210.82) | -7.158 | **<0.001** | **<0.001** | 164.19(141.71, 194.30) | 183.53(156.82, 213.32) | -7.472 | **<0.001** | **<0.001** |  |
| tyrosine | 88.63(69.05, 112.62) | 93.44(71.62, 116.70) | -2.556 | **0.011** | **0.019** | 91.21(74.05, 115.34) | 94.95(76.48, 116.96) | -1.887 | 0.059 | 0.082 |  |
| valine | 118.27(100.50, 140.54) | 127.24(108.13, 143.61) | -4.292 | **<0.001** | **<0.001** | 121.12(102.31, 144.54) | 130.17(112.54, 149.99) | -4.905 | **<0.001** | **<0.001** |  |
| tyrosine/phenylalanine ratio | 1.70(1.37, 2.15) | 1.84(1.44, 2.35) | -3.737 | **<0.001** | **<0.001** | 1.76(1.44, 2.29) | 1.86(1.48, 2.37) | -1.787 | 0.074 | 0.099 |  |
| ornithine/citrulline ratio | 9.55(8.08, 11.49) | 10.28(8.47, 12.83) | -4.935 | **<0.001** | **<0.001** | 9.43(7.63, 11.38) | 10.44(8.65, 12.44) | -5.789 | **<0.001** | **<0.001** |  |
| C0 | 22.90(18.97, 28.90) | 23.36(19.38, 28.30) | -0.525 | 0.600 | 0.641 | 21.43(17.35, 26.69) | 22.09(18.63, 26.66) | -2.208 | **0.027** | **0.041** |  |
| C2 | 19.90(15.95, 24.11) | 18.01(14.60, 21.77) | -5.366 | **<0.001** | **<0.001** | 18.12(14.79, 22.71) | 17.23(14.24, 20.61) | -3.704 | **<0.001** | **<0.001** |  |
| C3 | 1.61(1.26, 2.07) | 1.46(1.16, 1.86) | -4.703 | **<0.001** | **<0.001** | 1.50(1.18, 1.90) | 1.40(1.13, 1.82) | -2.613 | **0.009** | **0.018** |  |
| C3DC + C4OH | 0.08(0.06, 0.11) | 0.07(0.06, 0.10) | -4.892 | **<0.001** | **<0.001** | 0, 08(0.07, 0.11) | 0.07(0.06, 0.10) | -4.717 | **<0.001** | **<0.001** |  |
| C4 | 0.19(0.16, 0.23) | 0.19(0.16, 0.23) | -0.473 | 0.636 | 0.664 | 0.20(0.17, 0.24) | 0.20(0.17, 0.24) | -0.040 | 0.968 | 0.968 |  |
| C4DC + C5OH | 0.18(0.15, 0.21) | 0.18(0.15, 0.21) | -1.221 | 0.222 | 0.261 | 0.16(0.14, 0.19) | 0.17(0.15, 0.20) | -2.582 | **0.010** | **0.018** |  |
| C5 | 0.09(0.08, 0.11) | 0.10(0.08, 0.11) | -0.019 | 0.985 | 0.985 | 0.10(0.08, 0.13) | 0.10(0.09, 0.12) | -0.189 | 0.850 | 0.868 |  |
| C5:1 | 0.01(0.00, 0.01) | 0.00(0.00, 0.01) | -1.693 | 0.090 | 0.118 | 0.00(0.00, 0.01) | 0.00(0.00, 0.01) | -1.545 | 0.122 | 0.160 |  |
| C5DC + C6OH | 0.10(0.08, 0.12) | 0.09(0.07, 0.10) | -5.996 | **<0.001** | **<0.001** | 0.09(0.08, 0.11) | 0.09(0.07, 0.11) | -2.341 | **0.019** | **0.034** |  |
| C6 | 0.04(0.03, 0.05) | 0.03(0.03, 0.04) | -5.283 | **<0.001** | **<0.001** | 0.04(0.03, 0.04) | 0.03(0.03, 0.04) | -3.399 | **<0.001** | **0.002** |  |
| C6DC | 0.11(0.09, 0.15) | 0.12(0.09, 0.15) | -2.341 | **0.019** | **0.031** | 0.12(0.10, 0.16) | 0.13(0.10, 0.17) | -3.798 | **<0.001** | **<0.001** |  |
| C8 | 0.04(0.03, 0.06) | 0.04(0.03, 0.05) | -5.890 | **<0.001** | **<0.001** | 0.04(0.03, 0.05) | 0.04(0.03, 0.04) | **-7.847** | **<0.001** | **<0.001** |  |
| C8:1 | 0.11(0.09, 0.14) | 0.10(0.08, 0.12) | -5.373 | **<0.001** | **<0.001** | 0.11(0.09, 0.14) | 0.10(0.08, 0.12) | -5.749 | **<0.001** | **<0.001** |  |
| C10 | 0.06(0.04, 0.07) | 0.05(0.04, 0.06) | -4.862 | **<0.001** | **<0.001** | 0.05(0.04, 0.07) | 0.05(0.04, 0.06) | -6.545 | **<0.001** | **<0.001** |  |
| C10:1 | 0.06(0.05, 0.08) | 0.06(0.05, 0.08) | -1.872 | 0.061 | 0.085 | 0.06(0.05, 0.07) | 0.06(0.04, 0.07) | -3.329 | **<0.001** | **0.002** |  |
| C10:2 | 0.01(0.01, 0.01) | 0.01(0.01, 0.01) | -5.926 | **<0.001** | **<0.001** | 0.01(0.01, 0.01) | 0.01(0.01, 0.01) | -5.767 | **<0.001** | **<0.001** |  |
| C12 | 0.06(0.05, 0.08) | 0.05(0.04, 0.07) | -6.381 | **<0.001** | **<0.001** | 0.06(0.05, 0.07) | 0.05(0.04, 0.06) | -6.078 | **<0.001** | **<0.001** |  |
| C12:1 | 0, 05(0.03, 0.07) | 0.05(0.03, 0.07) | -0.334 | 0.738 | 0.755 | 0.04(0.03.0.06) | 0.04(0.03, 0.07) | -0.451 | 0.652 | 0.730 |  |
| C14 | 0.17(0.13.0.21) | 0.17(0.14, 0.20) | -2.037 | **0.042** | 0.059 | 0.15(0.12, 0.18) | 0.15(0.12, 0.18) | -0.778 | 0.436 | 0.513 |  |
| C14:1 | 0.08(0.06, 0.10) | 0.07(0.06, 0.09) | -4.061 | **<0.001** | **<0.001** | 0.07(0.06, 0.09) | 0.07(0.05, 0.08) | -1.925 | 0.054 | 0.080 |  |
| C14:2 | 0.02(0.01, 0.02) | 0.02(0.01, 0.02) | -3.953 | **<0.001** | **<0.001** | 0.02(0.01, 0.02) | 0.01(0.01, 0.02) | -4.352 | **<0.001** | **<0.001** |  |
| C14OH | 0.01(0.00, 0.01) | 0.01(0.00, 0.01) | -4.549 | **<0.001** | **<0.001** | 0.01(0.00, 0.01) | 0.01(0.00.0.01) | -4.455 | **<0.001** | **<0.001** |  |
| C16 | 2.38(1.84, 3.03) | 2.27(1.72, 2.90) | -2.296 | **0.022** | **0.034** | 2.29(1.71, 2.91) | 2.15(1.66, 2.66) | -2.914 | **0.004** | **0.007** |  |
| C16:1 | 0.11(0.08, 0.16) | 0.10(0.07, 0.14) | -3.592 | **<0.001** | **<0.001** | 0.11(0.08, 0.14) | 0.09(0.06, 0.12) | -4.728 | **<0.001** | **<0.001** |  |
| C16OH | 0.01(0.01, 0.01) | 0.01(0.01, 0.01) | -1.247 | 0.213 | 0.256 | 0.01(0.01, 0.01) | 0.01(0.01, 0.01) | -2.259 | **0.024** | **0.040** |  |
| C16:1OH | 0.03(0.03, 0.04) | 0.03(0.03, 0.04) | -1.337 | 0.181 | 0.224 | 0.03(0.02, 0.04) | 0.03(0.02, 0.04) | -0.390 | 0.696 | 0.759 |  |
| C18 | 0.73(0.58, 0.90) | 0.71(0.56, 0.89) | -1.130 | 0.259 | 0.289 | 0.73(0.57, 0.90) | 0.70(0.57, 0.85) | -1.886 | 0.059 | 0.082 |  |
| C18:1 | 1.37(1.10, 1.62) | 1.31(1.10, 1.54) | -2.237 | **0.025** | **0.038** | 1.27(1.04, 1.48) | 1.24(1.02, 1.46) | -1.066 | 0.287 | 0.354 |  |
| C18:1OH | 0.02(0.01, 0.02) | 0.01(0.01, 0.02) | -1.768 | 0.077 | 0.104 | 0.01(0.01, 0.02) | 0.01(0.01, 0.02) | -1.279 | 0.201 | 0.255 |  |
| C18:2 | 0.25(0.20, 0.32) | 0.25(0.19, 0.32) | -1.175 | 0.240 | 0.275 | 0.23(0.17, 0.29) | 0.23(0.18, 0.28) | -0.255 | 0.799 | 0.835 |  |
| C18OH | 0.01(0.00, 0.01) | 0.01(0.00, 0.01) | -3.452 | **<0.001** | **<0.001** | 0.01(0.00, 0.01) | 0.01(0.00, 0.01) | -3.463 | **<0.001** | **0.001** | |

Abbreviation: HDP: hypertensive disorders in pregnancy, FDR: false discovery rate, Leu\Ile\Pro-OH: leucine\isoleucine\hydroxyproline, C0: free carnitine, C2: acetylcarnitine, C3: propionylcarnitine, C3DC + C4OH: malonylcarnitine + 3-hydroxybutyrylcarnitine, C4: butyrylcarnitine + isobutyrylcarnitine, C4DC + C5OH: methylmalonyl + 3-hydroxy-isovalerylcarnitine, C5: sovalerylcarnitine + methylbutyrylcarnitine, C5:1:tiglylcarnitine, C5DC + C6OH: glutarylcarnitine + 3- hydroxyhexanoylcarnitine, C6: hexanoylcarnitine, C6DC: methylglutarylcarnitine, C8: octanoylcarnitine, C8:1: octenoylcarnitine, C10: decanoylcarnitine, C10:1: decenoylcarnitine, C10:2: decadienoylcarnitine, C12: dodecanoylcarnitine , C12:1:dodecenoylcarnitine, C14: ltetradecanoylcarnitine, C14:1: tetradecenoylcarnitine, C14:2: tetradecadienoylcarnitine, C14OH: 3-hydroxy-tetradecanoylcarnitine, C16: palmitoylcarnitine, C16:1: palmitoleylcarnitine, C16OH: 3-hydroxy-hexadecanoylcarnitine, C16:1OH: 3-hydroxy-hexadecenoylcarnitine, C18: stearoylcarnitine, C18:1: oleoylcarnitine, C18:1OH: 3-hydroxy-octadecenoylcarnitine, C18:2 :linoleoylcarnitine, C18OH: 3-hydroxy-octadecanoylcarnitine.

**Table S4. The FGR-stratified analyses of amino acid and acylcarnitine levels in peripheral blood of newborns in the hypertensive disorders in pregnancy group**

| **Amino acid or Acylcarnitine** | **HDP group** | | | | |
| --- | --- | --- | --- | --- | --- |
|  | **FGR(+)**  **(N=172)**  **Media (P25, P75)** | **FGR(-)**  **(N=1056)**  **Media (P25, P75)** | **Z-Value** | ***P*** | **FDR** |
| alanine | 296.48(239.85,364.31) | 299.10(249.64,353.61) | -0.286 | 0.775 | 0.852 |
| arginine | 6.74(4.23,12.21) | 8.61(5.07,14.62) | -2.768 | 0.006 | 0.019 |
| citrulline | 11.39(9.34,14.04) | 10.81(9.01,13.28) | -1.731 | 0.084 | 0.136 |
| glycine | 420.87(359.00,473.91) | 402.29(352.91,459.65) | -1.984 | 0.047 | 0.095 |
| Leu\Ile\  Pro-OH | 144.43(121.39,171.01) | 153.02(131.01,178.43) | -2.824 | 0.005 | 0.019 |
| methionine | 21.77(18.90,26.26) | 24.12(20.62,27.97) | -4.044 | <0.001 | 0.001 |
| ornithine | 96.89(77.33,119.443) | 103.73(85.65,125.33) | -2.816 | 0.005 | 0.019 |
| phenylalanine | 49.79(43.23,56.39) | 50.75(44.49,58.02) | -1.888 | 0.059 | 0.108 |
| proline | 159.12(133.33,192.56) | 164.40(142.61,191.91) | -2.166 | 0.030 | 0.080 |
| tyrosine | 87.96(66.69,114.01) | 89.96(72.81,114.42) | -1.269 | 0.205 | 0.300 |
| valine | 113.06(96.66,135.01) | 120.12(102.51,143.64) | -3.151 | 0.002 | 0.010 |
| tyrosine/phenylalanine ratio | 1.75(1.36,2.29) | 1.72(1.40,2.20) | -0.003 | 0.997 | 0.997 |
| ornithine/citrulline ratio | 8.75(7.10,10.19) | 9.73(7.99,11.57) | -4.513 | <0.001 | <0.001 |
| C0 | 25.09(20.09,31.40) | 21.84(18.09,27.24) | -4.517 | <0.001 | <0.001 |
| C2 | 20.70(16.69,26.26) | 18.68(15.22,22.95) | -3.757 | <0.001 | 0.002 |
| C3 | 1.49(1.16,1.87) | 1.57(1.23,2.02) | -1.671 | 0.095 | 0.149 |
| C3DC + C4OH | 0.09(0.07,0.12) | 0.08(0.06,0.11) | -1.891 | 0.059 | 0.108 |
| C4 | 0.20(0.16,0.24) | 0.20(0.17,0.23) | -0.379 | 0.705 | 0.795 |
| C4DC + C5OH | 0.16(0.14,0.20) | 0.17(0.15,0.20) | -2.127 | 0.033 | 0.080 |
| C5 | 0.10(0.08,0.12) | 0.10(0.08,0.12) | -0.124 | 0.902 | 0.945 |
| C5:1 | 0.00(0.00,0.01) | 0.01(0.00,0.01) | -0.750 | 0.453 | 0.554 |
| C5DC + C6OH | 0.09(0.08,0.11) | 0.09(0.08,0.11) | -0.260 | 0.795 | 0.853 |
| C6 | 0.04(0.03,0.05) | 0.04(0.03,0.05) | -1.246 | 0.213 | 0.302 |
| C6DC | 0.11(0.09,0.15) | 0.12(0.09,0.15) | -0.413 | 0.680 | 0.787 |
| C8 | 0.05(0.04,0.06) | 0.04(0.03,0.05) | -2.941 | 0.003 | 0.016 |
| C8:1 | 0.11(0.09,0.14) | 0.11(0.09,0.14) | -0.827 | 0.408 | 0.513 |
| C10 | 0.06(0.05,0.08) | 0.05(0.04,0.07) | -2.189 | 0.029 | 0.080 |
| C10:1 | 0.07(0.05,0.08) | 0.06(0.05,0.07) | -2.954 | 0.003 | 0.016 |
| C10:2 | 0.01(0.01,0.01) | 0.01(0.01,0.01) | -1.820 | 0.069 | 0.116 |
| C12 | 0.06(0.05,0.08) | 0.06(0.05,0.07) | -2.141 | 0.032 | 0.080 |
| C12:1 | 0.05(0.03,0.07) | 0.04(0.03,0.07) | -1.375 | 0.169 | 0.257 |
| C14 | 0.17(0.13,0.21) | 0.16(0.13,0.20) | -1.871 | 0.061 | 0.108 |
| C14:1 | 0.08(0.06,0.09) | 0.07(0.06,0.09) | -2.192 | 0.028 | 0.080 |
| C14:2 | 0.02(0.01,0.02) | 0.02(0.01,0.02) | -2.091 | 0.037 | 0.080 |
| C14OH | 0.01(0.00,0.01) | 0.01(0.00,0.01) | -0.062 | 0.951 | 0.973 |
| C16 | 2.46(1.80,3.14) | 2.32(1.76,2.96) | -0.879 | 0.380 | 0.491 |
| C16:1 | 0.11(0.08,0.16) | 0.11(0.08,0.15) | -1.206 | 0.228 | 0.313 |
| C16OH | 0.01(0.01,0.01) | 0.01(0.01,0.01) | -1.149 | 0.250 | 0.334 |
| C16:1OH | 0.03(0.03,0.04) | 0.03(0.02,0.04) | -2.073 | 0.038 | 0.080 |
| C18 | 0.78(0.62,0.94) | 0.72(0.57,0.89) | -2.083 | 0.037 | 0.080 |
| C18:1 | 1.39(1.18,1.66) | 1.29(1.06,1.54) | -3.495 | <0.001 | 0.003 |
| C18:1OH | 0.02(0.01,0.02) | 0.01(0.01,0.02) | -2.763 | 0.006 | 0.019 |
| C18:2 | 0.27(0.21,0.35) | 0.24(0.18,0.30) | -4.710 | <0.001 | <0.001 |
| C18OH | 0.01(0.00,0.01) | 0.01(0.00,0.01) | -0.509 | 0.611 | 0.726 |

Abbreviation: (+): with, (+): without, FGR: fetal growth restriction, HDP: hypertensive disorders in pregnancy, FDR: false discovery rate, Leu\Ile\Pro-OH: leucine\isoleucine\hydroxyproline, C0: free carnitine, C2: acetylcarnitine, C3: propionylcarnitine, C3DC + C4OH: malonylcarnitine + 3-hydroxybutyrylcarnitine, C4: butyrylcarnitine + isobutyrylcarnitine, C4DC + C5OH: methylmalonyl + 3-hydroxy-isovalerylcarnitine, C5: sovalerylcarnitine + methylbutyrylcarnitine, C5:1:tiglylcarnitine, C5DC + C6OH: glutarylcarnitine + 3- hydroxyhexanoylcarnitine, C6: hexanoylcarnitine, C6DC: methylglutarylcarnitine, C8: octanoylcarnitine, C8:1: octenoylcarnitine, C10: decanoylcarnitine, C10:1: decenoylcarnitine, C10:2: decadienoylcarnitine, C12: dodecanoylcarnitine , C12:1:dodecenoylcarnitine, C14: ltetradecanoylcarnitine, C14:1: tetradecenoylcarnitine, C14:2: tetradecadienoylcarnitine, C14OH: 3-hydroxy-tetradecanoylcarnitine, C16: palmitoylcarnitine, C16:1: palmitoleylcarnitine, C16OH: 3-hydroxy-hexadecanoylcarnitine, C16:1OH: 3-hydroxy-hexadecenoylcarnitine, C18: stearoylcarnitine, C18:1: oleoylcarnitine, C18:1OH: 3-hydroxy-octadecenoylcarnitine, C18:2 :linoleoylcarnitine, C18OH: 3-hydroxy-octadecanoylcarnitine.
